# Single-cell sequencing reveals effects of chemotherapy on the immune landscape and TCR/BCR clonal expansion in a relapsed ovarian cancer patient

**DOI:** 10.3389/fimmu.2022.985187

**Published:** 2022-09-28

**Authors:** Yanyu Ren, Runrong Li, Hanxiao Feng, Jieying Xie, Lin Gao, Shuai Chu, Yan Li, Fanliang Meng, Yunshan Ning

**Affiliations:** ^1^ School of Laboratory Medicine and Biotechnology, Southern Medical University, Guangzhou, China; ^2^ The First Clinical Medical School, Southern Medical University, Guangzhou, China; ^3^ Department of Clinical Laboratory, Nanfang Hospital, Southern Medical University, Guangzhou, China; ^4^ Department of Obstetrics and Gynecology, Nanfang Hospital, Southern Medical University, Guangzhou, China

**Keywords:** single-cell sequencing, ovarian cancer, chemotherapy, immune microenvironment, TCR/BCR repertoire, clonal expansion

## Abstract

Cancer recurrence and chemoresistance are the leading causes of death in high-grade serous ovarian cancer (HGSOC) patients. However, the unique role of the immune environment in tumor progression for relapsed chemo-resistant patients remains elusive. In single-cell resolution, we characterized a comprehensive multi-dimensional cellular and immunological atlas from tumor, ascites, and peripheral blood of a chemo-resistant patient at different stages of treatment. Our results highlight a role in recurrence and chemoresistance of the immunosuppressive microenvironment in ascites, including MDSC-like myeloid and hypo-metabolic γδT cells, and of peripheral CD8^+^ effector T cells with chemotherapy-induced senescent/exhaustive. Importantly, paired TCR/BCR sequencing demonstrated relative conservation of TCR clonal expansion in hyper-expanded CD8^+^ T cells and extensive BCR clonal expansion without usage bias of V(D)J genes after chemotherapy. Thus, our study suggests strategies for ameliorating chemotherapy-induced immune impairment to improve the clinical outcome of HGSOC.

## Introduction

Ovarian cancer (OC) is the ninth most common cause of cancer mortality in women and the second most common cause of gynecologic malignancy death worldwide (1). High-grade serous ovarian cancer (HGSOC), which is one of the most common and lethal pathological types of epithelial OC, poses a challenge to women’s health because of its recurrence and chemo-resistance. Platinum-based chemotherapy is the classical first-line treatment regimen for HGSOC and is usually effective initially. However, chemo-resistance eventually develops in about 70% of HGSOC patients after 3 years, leading to cancer relapse and eventually death (2, 3). Immune checkpoint blockade therapy has become a promising modality for a number of malignancies but shows limited benefits for HGSOC (4). Over the past two decades, primary cancer cells, malignant ascites, exfoliated cell clusters (also called “spheroids”) and immune cells have been identified in the unique tumor microenvironment (TME) of OC and are strongly associated with intra-abdominal distal organs metastasis, tumor relapse and diverse responses to drugs (5–7). Thus, investigation of the TME and its dynamic response to chemotherapy intervention is vital for elucidating the mechanisms underlying relapse and refractoriness of HGSOC.

Recently, scRNA-seq studies regarding HGSOC have clarified its origins and heterogeneity (8, 9), including its cellular landscape in ascites or metastatic loci (10–13), as well as the correlation between molecular subtypes and prognosis (14). However, several key points of understanding the impact of chemotherapy on HGSOC remain uncovered. First, the influence of chemotherapy on tumor tissue, ascites and PBMCs and the relationship between tumor cells and the TME remain elusive. Second, although the function and subtypes of T cells in HGSOC have been identified and shown to affect prognosis (10, 15), the role of B cells in HGSOC remains uncertain. Third, while V(D)J rearrangement is known to be the basis of immune system diversity that enables responses of T/B cells to antigens (16), dynamics of the TCR/BCR repertoire upon chemotherapy remains unclear in HGSOC. Finally, though the heterogeneity and function of macrophages in HGSOC ascites has been studied (10, 11), the myeloid cell shifts in the TME during platinum-based treatment have yet to be elucidated.

To this end, we utilized scRNA-seq and TCR/BCR sequencing to analyze the cancerous composition and immune community of a tumor lesion, malignancy ascites and peripheral blood from a chemotherapy-resistant relapsed HGSOC patient with progressively shorter progression-free survival (PFS) after several courses of platinum-based chemotherapy. We focused on the intrinsic features of tumor cells and explored the state of myeloid cells and T cells in the ascites. In peripheral blood mononuclear cells (PBMCs), we identified T/B cell subtypes and characterized the dynamics of the TCR/BCR repertoire upon chemotherapy. Our study provides insight into mechanisms of chemo-resistance from the aspect of immunity, thus providing fundamental evidence for implementing immunomodulatory therapies and improving treatment response in HGSOC.

## Materials and methods

### Collection of patient specimens and HGSOC scRNA-seq data

Specimens were collected from a patient with recurrent HGSOC at Nanfang Hospital. This study was approved by the Ethics Committee of Nanfang Hospital (NO. NFEC-2021-424). Informed consent was obtained from the patient prior to sample collection. During the second debulking surgery, solid tumor tissue was resected, washed in Dulbecco’s phosphate-buffered saline (DPBS, ThermoFisher Scientific, USA) and transported in DMEM solution. The ascites fluid was drained with a syringe and preserved in an aseptic 50 ml conical tube. Two specimens were transported on ice for further processing. After the surgery, the patient received the fourth course of platinum-based chemotherapy (six cycles), and PBMC samples were collected before and post this course of treatment. Identified patient information, including the ovarian cancer histology, stage, treatment history, Computed Tomography (CT) and Positron Emission Tomography-Computed Tomography (PET-CT) results, tumor markers and immunological indexes from peripheral blood were collected. 10X Genomics single-cell RNA sequencing data GSE154600 of five HGSOC patients (17), were download from GEO database (https://www.ncbi.nlm.nih.gov/geo/query/acc.cgi?acc=GSE154600). The dataset includes five HGSOC samples with different chemotherapy responses (*T59, T76, T77* chemo-resistant and *T89, T90* chemo-sensitive). 

### Preparation of single-cell suspensions

Within 6 hours after isolation, solid specimens were enzymatically dissociated into single cells. Briefly, the tissue was minced with a scalpel and enzymatically digested using 2 mg/mL Collagenase I (Worthington Biochemical) and 2 mg/mL Collagenase IV (Worthington Biochemical) for 30 minutes in a shaker (250 rpm) at 37°C. The digestion was terminated with DMEM + 5% fetal bovine serum (Thermo Fisher Scientific). The cell suspensions were sequentially filtered through 100 μm and then 70 μm cell strainers. Red blood cells were lysed by incubating the cell suspensions in RBC Lysis Solution (Sigma-Aldrich) for 3–10 minutes at 4°C. After centrifugation and resuspension, the concentrations of the single-cell suspensions were adjusted to 7–12×10^5^ cells/ml with 5% fetal bovine serum DMEM. Ascites was centrifuged for 10 min at 4°C, and the remaining pellet was resuspended in PBS, filtered, subjected to RBC lysis, and resuspended as described for the tumor samples. Peripheral blood was collected into heparin tubes (Becton, Dickinson and Company) and processed within 2 hours of collection. PBMCs were isolated by density gradient centrifugation using Ficoll-Paque Plus medium and washed with Ca/Mg-free PBS. The isolated cells derived from above samples were used for single cell sequencing.

### Preparation of single cell RNA-seq, TCR-seq and BCR-seq libraries

The suspensions of live cells in sterile-filtered PBS (Corning) with 0.04% BSA (Sigma Aldrich) were used as input for the 10× Chromium controller system (10× Genomics Inc.). Using 10× GemCode Technology, the cells were barcoded to separately index each cell’s transcriptome by partitioning them into Gel Bead-in-EMulsions (GEMs). Barcoded Single Cell 50 Gel Beads, RT Master Mix with cells and Partitioning Oil were combined on a microfluidic chip, and GEMs were generated. The GEM RT reactions were activated in a thermocycler (53°C for 45 min, 85°C for 5 min, 4°C hold overnight). Post RT incubation, the GEMs were disrupted and the first-strand cDNA was recovered. cDNA amplification was performed by PCR to generate sufficient material. According to the manufacturer’s instructions, scRNA-seq libraries of tumor tissue and ascites were generated using Chromium™ Single Cell 3’ Library (v3 chemistry) reagents. For PBMC samples, scRNA-seq libraries were processed using the Chromium™ single cell 5’ library & gel bead kit and coupled TCR/BCR libraries were obtained using the Chromium™ single cell V(D)J enrichment kit (10× Genomics). Libraries of scRNA-seq were sequenced on the Illumina Novaseq 6000.

### Immunohistochemistry staining

The tumor tissues were collected from Nanfang Hospital. IHC staining was carried out with anti-CD8 antibody (18187-1-AP, 1:200 dilution; Proteintech, Rosemont, USA). The immunostaining results were examined independently by two researchers. Paraffin-embedded ovarian cancer tissues were cut into 4 µm thick sections. Histological evaluation was done with hematoxylin and eosin (H&E). Immunohistochemical staining was performed to confirm the presence of CD8 cells. Briefly, sections were deparaffinized and rehydrated using xylene and serial dilutions of EtOH in distilled water. Tissue sections were incubated in citrate buffer, pH 6 and heated in a microwave oven. Anti-CD8 (18187-1-AP, 1:200 dilution; Proteintech, Rosemont, USA) antibody was applied on tissue sections with one-hour incubation at room temperature in a humidity chamber. Antigen-antibody binding was detected with the labeled polymer-HRP Envision system (DAKO, K4007) and DAB+ chromogen (DAKO, K3468) system. Tissue sections were briefly immersed in hematoxylin for counterstaining and were covered with cover glasses.

### Single cell seq data processing

Pre-processing of scRNA-seq fastq files was conducted using Cell Ranger v4.0.0 (10× Genomics). ScRNA-seq reads were aligned to GRCh38, and a count matrix of cell barcodes for downstream analysis was generated using the Cell Ranger count function with parameter–expect-cells = 3000. The raw count matrix for each sample was obtained from the Cell Ranger count filter matrix output (18). The pipeline generates a UMI count matrix, which is processed using Seurat software (4.0.5). Integrated analysis of multimodal single-cell data was achieved using previously established methods (19). The quality of cells was assessed based on three metrics parameters to remove low-quality cells and multiple-like microdropletsCells meeting the following criteria are reserved: (1) The number of total UMI counts per cell (≥500); (2) The number of detected genes per cell (≥500); and (3) The proportion of mitochondrial genes (≤25%). The remaining cells were subjected to further analyses.

### Integration, dimension reduction and unsupervised clustering

Core scRNA-seq analysis was performed using Seurat v4.0.5. The counts for each library were normalized using the NormalizeData function. The most highly variable genes were selected using the FindVariable function in Seurat and a PCA matrix with 20 components employing variable genes by using the RunPCA function implemented in the Seurat package. To integrate datasets into a mutual space from different tissues for unsupervised clustering, we used the harmony algorithm, followed by PCA-reduced dimensionality. Then, the mutual nearest neighbor (MNN) was calculated. The shared nearest neighbor (SNN) algorithm, which is the default algorithm for clustering in the pipeline of Seurat, was used for clustering. It includes two steps corresponding to the two functions. First, FindNeighbors was used to calculate the K-nearest neighbors (KNN) of each cell and construct the SNN graph image. Second, FindClusters was used to find cell clusters according to the SNN graph results (“graph-based clustering”). Cells were reclustered separately according to specified parameters without engaging the other cell types. After clustering based on gene expression patterns employing the FindClusters function, cells were visualized with the RunTSNE function in Seurat. Cluster identification was performed at a resolution that best separated the different cell types. Clusters were annotated based on the expression of known marker genes of each cell type. To identify clusters within each major cell type, we performed a second round of clustering for specified cell populations. To discover the relationship among specific samples, the expression matrix was integrated, clustered, and annotated again. The procedure of each round of clustering was the same as the first round, starting from the expression matrix, including finding the most highly variable genes, calculating the PCA matrix, as well as performing integration analysis using the harmony algorithm, dimensional reduction and unsupervised clustering analysis by Seurat.

### Identification of differentially expressed genes and gene set enrichment analysis

We applied the FindAllMarkers function (test. use = Wilcox) in Seurat to identify marker genes of each cluster. For a given cluster, positive markers were compared with other cell groups. The signification threshold was set as *P*<0.05 and |log_2_ foldchange|>0.25. GO (Gene Ontology Enrichment Analysis) and KEGG analyses of differentially expressed genes were conducted using R package clusterProfiler (20). Specific gene sets were obtained from the Molecular Signature Database (MSigDB; https://www.gsea-sigdb.org/gsea/downloads.jsp). To characterize subclusters of epithelial cells (tumor cells), we performed single-sample gene set enrichment analysis (ssGSEA) of 50 hallmark gene sets (h.all.v7.1.symbols.gmt downloaded from MSigDB) for each subcluster and single cells using R package GSVA. Heatmaps were used to display the results of GSVA based on the average expression, and violin plots were used to display the pathway enrichment results based on the expression of each tumor cell. The pre‐ranked gene set enrichment analysis method (R package fgsea) that was designed for GSEA of single-cell sequencing was also conducted to compare functional differences in macrophage populations between the tumor and ascites samples. Genes were ranked by the average log‐fold change calculated by the FindMarkers function in Seurat.

### G2/M phase identification

For G2/M phase analysis in the tumor compartment, we calculated a G2/M score for each tumor cell using the CellCycleScoring function in Seurat. The per-cell scores were added to the metadata matrix to assess the cell phase of the subclusters in tumor cells, and the stage of the cell phase of each cell was displayed as a t-SNE plot.

### Trajectory analysis of single cells

We used the R package Monocle2 (v2.20.0) to perform pseudotime analysis to project high-dimensional transcriptomic data to one dimension that characterizes the relationship between monocytes and macrophages from tumor tissue, ascites and PBMCs. The matrix in the scale of raw UMI counts derived from Seurat objects were converted into new objects by the newCellDataSet function. Genes with mean expression ≥0.1 were used in the trajectory analysis. Selected genes with q-value < 0.01 between the cell groups were applied for dimensional reduction using the reduceDimension function with the parameter reduction_method= “DDRTree” and max_components=2. The trajectory plots were visualized using the plot_cell_trajectory function.

### CellChat analysis

Cell communication analysis was performed between epithelial cells and macrophages in tumor and ascites tissues. R package CellChat (v1.1.2), which contains ligand-receptor interaction databases, analyze the intercellular communication networks between different cell clusters from scRNA-seq data (21).First, CellChat was used to evaluate the major signaling inputs and outputs among all epithelial cells and macrophages subclusters in tumor tissue. Next, netVisual_bubble function was utilized to show the significant ligand-receptor interactions between subclusters included.

### inferCNV analysis

CNVs analysis of six tumor samples were performed by R package inferCNV(v1.8.1). The inferCNV cutoff parameter was set to 0.1 and HMM option was set to TRUE. The CNVs of tumor cells were calculated by raw expression data compared to myeloid subclusters from each sample. For inferCNV, 400 cells per subcluster were pseudorandomly chosen. CNVs values of each cell were finally limited as -1 to 1. The CNVs score of each cell was calculated as quadratic sum of CNV region.

### Flow cytometry analysis

Flow cytometry analysis on patient peripheral blood samples was conducted at three times during the fourth cycle of chemotherapy (T1: before the second chemotherapy began; T2: two days after the sixth chemotherapy; T3: fourteen days after the sixth chemotherapy). Single cell suspensions were stained with antibodies for surface markers. Cells were washed and resuspended in FACS Buffers (PBS+0.5% HI-FBS) until data collection. Flow cytometry was performed with LSR II flow cytometer (BD Bioscience), and data analysis was conducted by FlowJo software. MultitestTM 6-color TBNK (Cat:644611), Fluorescein isothiocyanate (FITC)-conjugated anti-CD4 (Cat:340133), Fluorescein isothiocyanate (FITC)-conjugated anti-CD3 (Cat:349201), allophycocyanin (APC)-conjugated anti-CD25 (Cat:662525), chlorophyll protein complex-(PerCP)-conjugated anti-CD3 (Cat:652831), chlorophyll protein complex-(PerCP)-conjugated anti-CD45 (Cat:561047), allophycocyanin (APC)-cyanine 7-conjugated anti-CD4 (Cat:341115), phycoerythrin (PE)-cyanine 7-conjugated anti-CD8(Cat:1292923), phycoerythrin(PE)-conjugated anti-CD25(Cat:652834), Fluorescein isothiocyanate (FITC)-conjugated anti-CD45RA (Cat:662840), and hemolysin for flow cytometry were purchased from BD Biosciences, USA. Absolute number of tubes were purchased from BD Biosciences, USA. Phycoerythrin (PE)-conjugated anti-CD127 (Cat: P010034-B) were bought from Jiangxi CELGENE Biotechnology corporation, P.R. China.

### Cytokine assay

Interleukin level assessment on patient peripheral blood samples was conducted at three times during the fourth cycle of chemotherapy (before the second chemotherapy began, two days after the sixth chemotherapy and fourteen days after the sixth chemotherapy). Utilizing an ELISA kit (Biosource, Invitrogen, USA) in accordance with the manufacturer’s instructions, inflammation markers including tumor necrosis factor-α(TNF-α), interferon-gamma (IFN-γ), interleukin-2 (IL-2), interleukin-4 (IL-4), interleukin-6 (IL-6), interleukin-10 (IL-10) and interleukin-17 (IL-17) concentrations were measured.

### TCGA data analysis

We evaluated the function of core IFN-associated genes (obtained from hallmark gene sets (h.all.v7.1.symbols.gmt) of MSigDB in HGSOC. The TCGA ovarian carcinoma (OV) data were used to predict the correlation of selected genes and patient survival. The gene expression data (counts matrix) and the clinical data were downloaded from UCSC Xena (http://xena.ucsc.edu/). Transcriptional matrices with paired clinical data were selected for analysis. Signatures were dichotomized into high-expression and low-expression groups based on the median GSVA values of per TCGA sample. Quartiles were plotted using R packages survival and survminer, and the p-value of the IFN signatures was calculated using the log-rank test.

### Processing of single cell TCR and BCR sequencing libraries

The TCR and BCR sequences for T/B cells were collected from single-cell RNA-Seq data provided by 10× Genomics. Gene quantification and TCR/BCR clonotype assignment were performed using Cell Ranger (v.4.0.0) vdj pipeline with GRCh38 as reference. In this way, we obtained a TCR/BCR diversity metric, containing clonotype frequency and barcode information.

For the TCR, cells with no obvious TCR forms were excluded first, and TCR α/β chains were then obtained with reference to previous work (22). The target TCR α/β chains were defined as follows: (1) TCR barcodes could be found in T cells population from single cell mRNA sequencing data; (2) TCR with only one productive TCR α and β chain were retained. If multiple TCR α or β chains were identified in a T cell, only cells with dominant forms of α and β were retained.

For the BCR, similar filtration steps were conducted. Only cells with productive, paired heavy chain (IGH) and light chain (IGK or IGL) were reserved. After filtration, there were 6201 TCR-positive T cells and 1631 BCR-positive B cells in two blood samples.

### Single cell TCR and BCR clonotype analysis

Clonotype analysis of TCR was conducted using the scRepertoire toolkit (23) based on TCR-seq libraries. Each unique TRA(s)-TRB(s) pair was defined as a clonotype in TCR, while each unique IGH(s)-IGK/IGL(s) pair was considered as a clonotype in BCR. If one clonotype was present in at least two cells, the cells possessing this clonotype were regarded as clonal.

For TCR and BCR clonotype analyses, the clonal homeostasis and clonal space occupied by clonotypes of specific proportions were first identified, and the proportion of clonal space occupied by specific clonotypes was visualized using the *clonalHomeostasis* and *clonalProportion* functions. Next, using the *clonalDiversity* function, the diversity across cell clusters was measured using Shannon, Inverse Simpson, Chao, and abundance-based coverage estimator (ACE) indices. Based on the *clonalOverlap* function, the clonotype overlap between cell clusters was then calculated and visualized using *Morisita* index methods. With *quantContig* function, unique clonotypes were scaled to the size of the sample library. Furthermore, the distribution of CDR3 amino acid sequences (whole, TRA, and TRB) was then identified using the *lengthContig* function. Moreover, we chose the top ten most-expanded clonotypes as dominant clonotypes and used *alluvialClonotypes* function to examine their dynamics in T/B cells after chemotherapy.

### Single-cell TCR/BCR V(D)J sequencing and analysis

V(D)J sequence assembly, and paired clonotype calling was performed using CellRanger vdj with -reference = refdata-cellranger-vdh-GRCh38-atlas-ensembl-4.0.0 for each sample. Subsequent work was conducted based on the basic statistic function in R. We first calculated the usage of TRAV/J, TRBV/J, IGHV/J and IGLV/J gene segments. Next, we identified the percentage of each gene segments used. V-J pairs of TCR α/β chains and corresponding frequencies were later confirmed. For the CDR3 amino acid (aa) length, we measured the frequency of TCR/BCR segments with the same aa length, to explore the distribution of the CDR3 aa length.

### Survival analysis

Analysis of the association of interferon-associated genes with survival times in TCGA-OV datasets downloaded from UCSC Xena was conducted using the Survival Package, and p-values were calculated using the log-rank test.

### Statistical analysis and data visualization

All statistical analyses were performed in software R. Significance was defined as a p value less than 0.05. The Wilcox-test in the Findmarker function in Seurat was performed to distinguish differential expressed genes between different clusters. Pairwise wilcoxon tests were calculated to compare the expression of specific genes between different samples or cell subclusters. The usage bias of V(D)J genes in TCR/BCR was identified by FDR (adjusted p values) using the Fisher test (< 0.05). Clinical statistical analyses (**Supplementary Figure S6**) were visualized using Graphpad PRISM (version 8.1.0).

## Results

### The cellular composition of a solitary lesion and ascites from a relapsed chemo-resistant ovarian cancer patient

**Figure 1:**
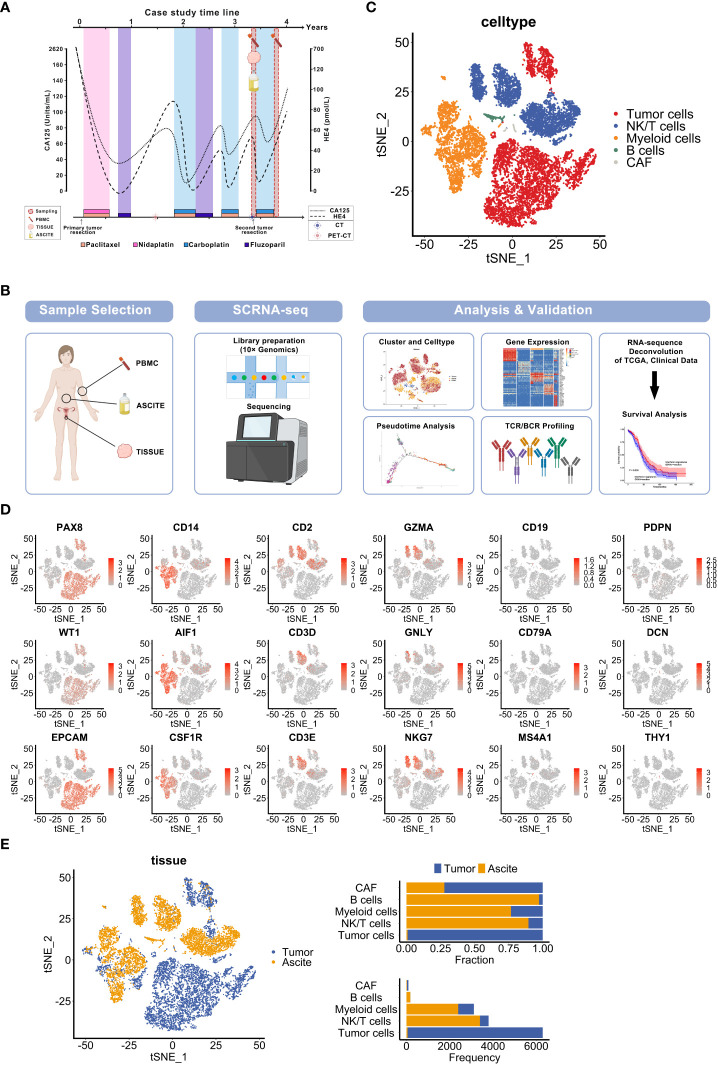
Single-cell sequencing to characterize the diverse cellular components of specimens derived from a recurrent ovarian cancer patient. **(A)** Overview of the clinical course and sample collection time of an HGSOC patient. The curved lines indicate changes of tumor biomarkers (CA125, HE4). The timepoints of chemotherapy treatment are shown in the label. **(B)** Overview of the sample collection, profiling strategy and analysis workflow. **(C)** t-SNE visualization of diverse cell types in sample *Tumor* and *Ascite*, colored by each cell type. **(D)** t-SNE plots show cell-type marker genes expression level. **(E)** t-SNE visualization of cells from samples *Tumor* and *Ascite*, colored by sample origin (left panel). Fraction and frequency of cells (x axis) from tumor tissues and ascites in each cell type (y axis) (right panel).

To elaborate the characteristics of ovarian cancer patients who experience a gradual transition from chemo-sensitivity to -resistance and repeated tumor recurrence despite having received standard and extensive treatment, we evaluated a stage IIIC HGSOC patient. The patient initially underwent primary optimal surgical debulking followed by paclitaxel combined with nedaplatin and experienced the first recurrence after 17 months, indicating platinum-sensitive recurrent relapsed ovarian cancer (24). Unfortunately, she experienced three additional relapses indicated by re-ascending serum CA125/HE4 and imaging, and her PFS became shorter within each recurrence (from 7 months to 4 months to 2 months), suggesting that she developed chemo-resistance (**Figure 1A**). After three complete courses of platinum/taxol-based chemotherapy, she encountered a third relapse and accepted secondary cytoreductive surgery. To dissect the cellular composition and function of the TME at a key transient period from chemo-sensitiveness to chemo-resistance, as well as the impact of multi-cycles chemotherapy on the immune system, we collected a solitary mass from the vaginal cuff, peritoneal cavity ascites, and PBMCs for further study, with informed consent from the patient and approval of local institutional ethical review board.

To characterize the cellular components of these samples, we generated and analyzed single-cell transcriptomic profiles using 10× Genomics platform (**Figure 1B**). Based on known cell type markers, we identified and classified 5 cell types displayed by *t*-distributed stochastic neighbor embedding (t-SNE) as follows: tumor cells (*EPCAM, PAX8, WT1*), myeloid cells (*CD14, AIF1, CSF1R*), NK/T cells (*CD2, CD3E, CD3D, GZMA, GNLY, NKG7*), B lymphocytes (CD19, *CD79A, MS4A1*), and cancer-associated fibroblasts (CAFs) (*PDPN, DCN, THY1*) (**Figures 1C, D**). Similar with previous HGSOC single-cell sequencing reports (8, 11, 25), epithelial cancer cells were the most abundant cellular components followed by myeloid cells in tumor tissues. Contrary to previous research (8), immune cells dominates in ascites but rarely are found in the tumor in our study. CAFs were sparse in both the tumor tissue and ascites (**Figure 1E**). Moreover, T cells were less abundant in the tumor compared with the ascites (**Figure 1E**). Thus, these findings are suggestive of potential roles for both epithelial cells and immune cells in recurrence.

### Functional and biological features of epithelial tumor cells from the relapsed lesion or ascites

**Figure 2:**
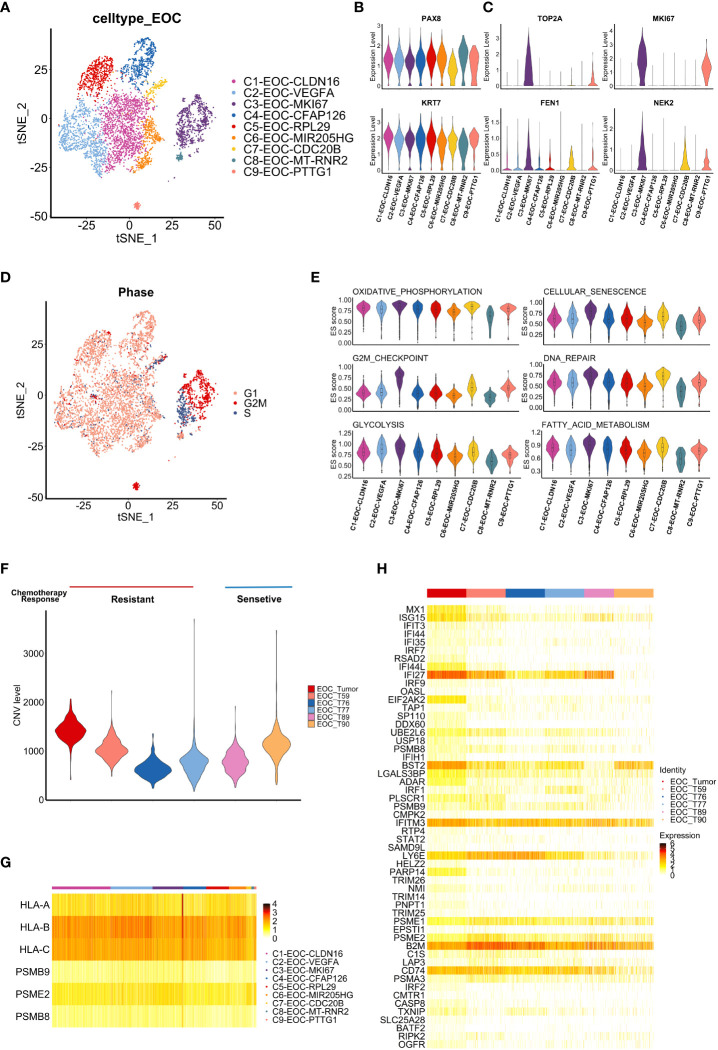
Tumor-intrinsic features uncovered by single cell analysis. **(A)** t-SNE visualization of tumor cell subclusters from samples *Tumor* and *Ascites*, colored by tumor cell subclusters. **(B, C)** Violin plots display the expression of fallopian tube epithelium (FTE) markers **(B)** and chemotherapy resistance-related genes **(C)** in each tumor cell subclusters. Distribution of the per cell signature expression Is based on normalized data. **(D)** t-SNE visualization of cell cycle phases of tumor cells in sample *Tumor* and *Ascites*, colored by cell cycle phase. **(E)** Violin plots shows the enrichment level of specific pathways among each tumor cell subcluster. Distribution of the per cell signature expression was based on the GSVA scores. **(F)** Violin plot shows CNV level among tumor cells from our data and five additional HGSOC samples. (*Tumor, T59, T76*, *T77*: chemo-resistant; *T89*, *T90*: chemo-sensitive) **(G)** Heatmap shows the expression of antigen presentation related genes in tumor cell subclusters from sample *Tumor*. **(H)** Heatmap shows the expression of interferon response pathway-associated genes in tumor cells from six HGSOC samples (*Tumor, T59, T76, T77, T89, T90*).

We next analyzed the inherent features of cancer cells from the relapsed solitary tumor and ascites. Nine clusters of epithelial malignancy cells were identified (**Figure 2A** and **Supplementary Figure S1A**). The fallopian tube epithelium (FTE) markers *PAX8* and *KRT7* (9) were overexpressed in all subclusters, suggesting that the tumor may originate from FTE (**Figure 2B**). Furthermore, the C3-EOC-MKI67 (EC3) subpopulation displayed higher expression of chemotherapy resistance-related genes (*FEN1, NEK2, TOP2A* and *MKI67)* (**Figure 2C**) (8). Using the CellCycleScoring function of Seurat, we determined that the EC3 subgroup of cells were mainly in the S and G2/M phases, indicating that they were characterized by hyperproliferative status (**Figure 2D**). To functionally annotate the malignant epithelial clusters, we conducted Gene Set Variation Analysis (GSVA) based on hallmark gene sets from Molecular Signatures Database (MSigDB) (**Supplementary Figure S1B**). The EC3 population exhibited relatively higher enrichment in several pathways, including oxidative phosphorylation, cellular senescence, G2/M checkpoint, DNA repair pathways, glycolysis, and fatty acid metabolism (**Figure 2E**). These results suggest that EC3 cells, with features of hyperproliferation, hypermetabolism and chemo-resistance, may account for the progression and recurrence of ovarian cancer.

To validate our findings, scRNA-seq datasets of five HGSOC patients (*T59, T76, T77, T89, T90*) were downloaded from GEO database (GSE154600) (17), which contains respective chemotherapy response. After the integration, dimension reduction and unsupervised clustering mentioned in methods, the same cell types were identified (**Supplementary Figure S1C**). Tumor cells in chemo-resistant samples showed higher expression of chemoresistance and proliferation related genes (*FN1, LCN2, CD44, FEN1*) (**Supplementary Figure S1D**) (8, 26–28). Given the association between the malignant tumor and large-scale chromosomal alterations, copy-number variation (CNV) of epithelial ovarian cancer (EOC) cells in six samples were contrasted with myeloid cells (**Supplementary Figure S1E**). Result of CNV analysis showed that our sample (EOC_Tumor) displayed elevated CNV levels (**Figure 2F**). Moreover, we explore whether the EC3 cluster is a characteristic cluster in chemo-resistant tumors. The top 10 expressed genes in this cluster were selected to assess the correlation among six samples (**Supplementary Figure S1F**). Results showed that our sample (EOC_Tumor) harbored higher similarity with two chemo-resistant samples (*T76* and *T77*) in expression profile contrast to two chemo-sensitive samples (*T89* and *T90*) (**Supplementary Figure S1G**). In conclusion, these results suggest that MKI67 positive cancer cells may contribute to chemotherapy resistance in HGSOC.

Next, we evaluated the expression of antigen presentation-related genes in cancer cells. Similar with the previous report (10), *HLA-B* and *HLA-C* had commonly obvious expression among subclusters (**Figure 2G**). Furthermore, interferon (IFN) pathway-associated genes were uniformly enriched among most subclusters of tumor cells. Genes associated with the IFN response (e. g. *IFI27, IFITM3, LY6E*), which represents core genes of the IFN pathway, were significantly elevated in tumor cells (**Supplementary Figure S1H**). To validate our findings, we characterized the expression of these genes in GEO database (GSE154600) and obtained the similar expression profile (**Figure 2H**). To further predict potential functions of IFN-associated genes in HGSOC, we performed survival analysis based on these genes using the OV-TCGA dataset, which suggested that high expression of IFN-associated genes is related to a better prognosis (log-rank method, *P*= 0.039) (**Supplementary Figure SI**). Thus, enrichment of the IFN expression profile (**Figure 2H**) in relapsed tumor may suggest stronger immune response and good prognosis in this patient. However, the progressively shorter PFS3 of this patient calls for further investigation on tumor immune microenvironment (TIME).

### Dissection of the components and role of myeloid cells in TIME

To better elucidate TIME, we analyzed 17301 immune cells from samples collected before the fourth course of chemotherapy, including 910 cells from the tumor, 5949 cells from ascites and 10442 cells from PBMCs. Four major cell types were identified based on previously characterized markers (8, 10), including T cells (*CD3D, CD3G, CD2*), NK cells (*NKG7, GNLY, KLRD1, KLRF1*), B cells (*MS4A1, CD19, CD79A, CD79B*) and myeloid cells (*CD14, AIF1, CSF1R*) (**Supplementary Figures S2A–C**). T cells and myeloid cells were the dominant immune cells in the ascites and tumor (**Supplementary Figure S2D**), which is consistent with other studies (8, 10, 29).

**Figure 3:**
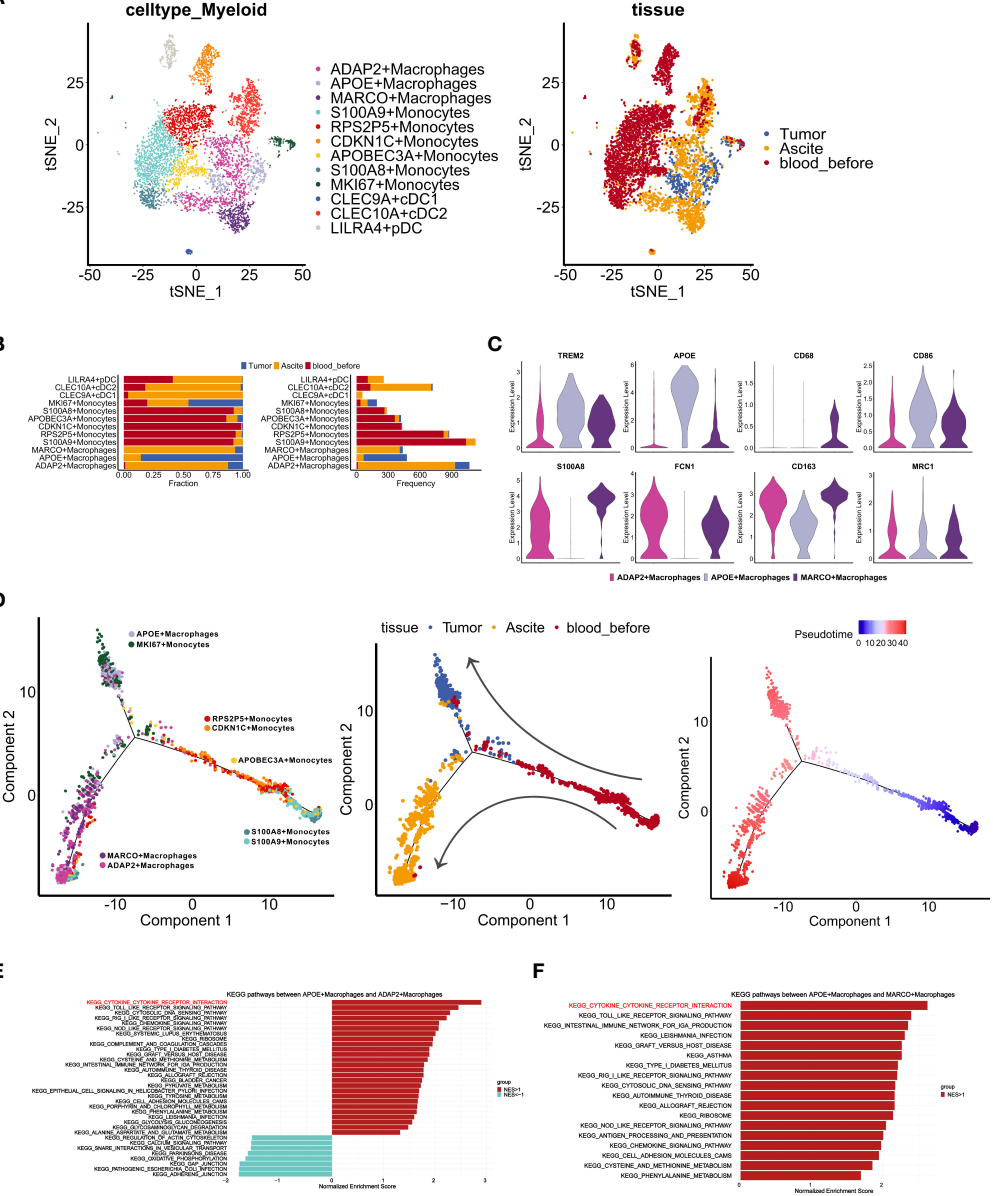
Characteristics of myeloid cells in distinct TMEs of ascites, tumor and PBMCs. **(A)** t-SNE visualization of myeloid cells profiles from three samples (*Tumor, Asicite, blood_before*) before the fourth course of chemotherapy, colored by myeloid cell subclusters (left panel) and sample origins (right panel). **(B)** Fraction and frequency of myeloid cells (x axis) from samples (*Tumor, Asicite, blood_before*) in each myeloid subcluster (y axis). **(C)** Violin plots display the expression TAM- (*TREM2, APOE*), MDSC- (*S100A8, FCN1*) and M1-like (*CD68, CD86*), M2-like (*CD163, MRC1*) signatures expression among three macrophage subclusters (ADAP2+ Macrophages, APOE+ Macrophages, MARCO+ Macrophages). **(D)**Pseudotime analysis of monocytes and macrophages from samples (*Tumor, Asicite, blood_before*), colored by each myeloid subcluster (left panel), derived-samples (middle panel) and pseudotime trajectory (right panel). **(E, F)** Gene set enrichment analysis between APOE subcluster and ADAP2 subcluster **(E)**, APOE and MARCO subcluster **(F)** using KEGG gene sets. Pathway enrichment is expressed as normalized enrichment score (NES).

We next performed cluster analysis of the myeloid cells and revealed 12 clusters (**Figure 3A**). Based on previous report (30), we applied genes predominantly expressed in blood-derived monocytes (*S100A8, S100A9 and CSF3R*) and classical monocytes markers (*CD14, CD16 and FCN1*) together as monocytes markers. Consistently (30), high expression of these six markers in monocytes reflect that monocytes are probably educated by TIME. The cluster populations were primarily comprised of six monocyte clusters with high expression of *S100A8, S100A9, RPS2P5, CDKN1C*, and *MKI67*, and three macrophage clusters with high expression of *ADAP2, MARCO* and APOE (**Supplementary Figures S2E, F**). Of note, MKI67 monocytes and APOE macrophages were mainly derived from tumor, while ADAP2 and MARCO macrophages were mainly from ascites (**Figure 3B**). Using markers identified in a previous report (31), we found that APOE macrophages exhibited TAM-like signatures (*TREM2, APOE*), whereas ADAP2 macrophages highly expressed MDSC-like signatures (*S100A8, FCN1*) (**Figure 3C**). In addition, these two clusters showed high expression of M2-like signatures (*CD163, MRC1*), while ascites-derived MARCO macrophages highly expressed MDSC-like signatures with both M1- (*CD68, CD86*) and M2-like signatures (**Figure 3C**). Next, we explored the trajectory of myeloid cells from different sites by pseudo-time analysis. Except for MKI67 monocytes, PBMC-derived monocytes bifurcated to ascites-resident macrophage populations (ADAP2 and MARCO macrophages) and tumor-resident populations (MKI67 monocytes and APOE macrophages) (**Figure 3D**), suggesting that peripheral monocytes may migrate to ascites and tumors, and be educated as different subtypes in the TIME.

To characterize the different functions of macrophages in the ascites and tumor, we compared KEGG pathways that were enriched in different subpopulations. Compared with the APOE cluster, both the ADAP2 and MARCO clusters showed lower enrichment of cytokine receptor interactions (**Figures 3E, F**), indicating impaired activation and cytotoxicity of macrophages in ascites. Moreover, we investigated expression of CCL/CXCL ligand in tumor clusters (**Supplementary Figures S3A, B**) and CCR/CXCR receptors in myeloid clusters (**Supplementary Figures S3C, D**). *CXCL16*-*CXCR6*, known tumor cell-immune cell crosstalk in immune infiltrated tumors (10), showed rare co-expression (**Supplementary Figures S3B, D**), suggesting the lack of immune cells recruitment mediated *via CXCL16*. To further inspect the interaction between tumor cells and myeloid cells, we performed communication analysis using R package CellChat. We observed top-ranking ligand-receptor pairs of macrophage migration inhibitory factor (MIF) in cancer cells and (CD74+CD44) in macrophages (**Supplementary Figures S3E, F**). Contributing to anti-inflammatory, and immune evasive phenotypes in malignant disease (32), MIF was also reported to be elevated in ovarian cancer cells (33). In addition, Midkine (MDK)-LRP1 pairs, which promotes immunosuppressive macrophage differentiation (34), markedly exist from epithelial clusters to macrophages (**Supplementary Figures S3E, F**). Together, these results suggest an immune-suppressive state of macrophages in this patient.

In order to confirm whether above characteristics in TIME are unique to drug-resistant tumors, we integrated GSE154600 and our data to identify T cells, B cells, and myeloid cells in all samples. The results showed that proportion of myeloid cells was higher in chemo-resistant tumors, especially in our sample (**Supplementary Figure S3G**). Myeloid cells were selected for further clustering and macrophages, monocytes and DC cells were identified (**Figure S3H**). As expected, expression of the M2 signatures (*CD163, MRC1*) was higher in chemo-resistant samples while M1 signatures (*CD68, SOCS3*) (35) expression was low. Immune-suppressive genes (*GPNMB*, *TREM2*) (36) had elevated level in chemo-resistant samples as well (**Supplementary Figure S3I**). In summary, these results indicate that macrophages with immune-suppressed phenotype may be a character of chemo-resistant HGSOC.

### The inhibitory status of γδT cells contributes to the immunosuppressive environment in ascites

**Figure 4:**
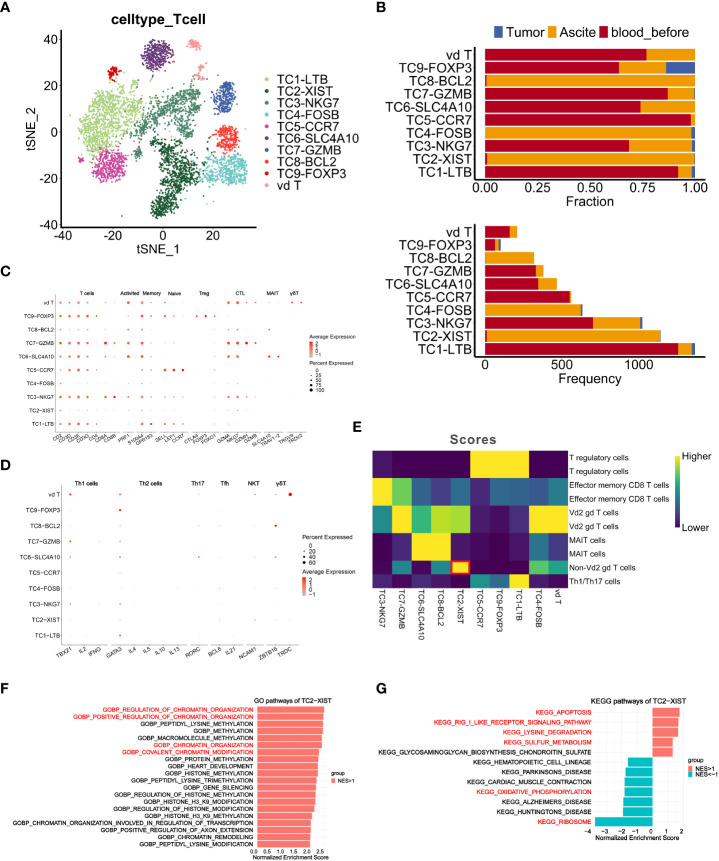
Characteristics and dynamics of T cells from samples before chemotherapy at single-cell resolution. **(A, B)** t-SNE visualization of T cell clusters from samples (*Tumor, Ascite, blood_before*) before chemotherapy, colored by the identified T cell subclusters. **(B)** Fraction and frequency of T cells (x axis) from samples (*Tumor, Ascite, blood_before*) in each T cell subcluster (y axis). **(C, D)** Dot plots show expression level of signature genes in each T cell subcluster. **(E)** Heatmap shows the classification result of each T cell subclusters using singleR. **(F, G)** Gene set enrichment analysis of T cell subcluster TC2-XIST using GO gene sets **(F)** and KEGG gene sets **(G)**. Pathway enrichment is expressed as normalized enrichment score (NES).

To clarify the role of T cells in TME, we clustered T cells based on the expression of surface markers of cells from tumor, ascites, and PBMCs (**Figures 4A, B**). Seven T cell clusters were characterized as follows: activated T cells (*PRF1*), memory T cells (*S100A4, GPR183*), naïve T cells (*SELL, LEF1, CCR7*), Tregs (*CTLA4, FOXP3, FOXO1*), cytotoxic T lymphocytes (CTL) (*GZMA, NKG7, GZMH, GZMB*), mucosal-associated invariant T cells (MAIT) (*SLC4A10, TRAV1-2*) and γδT cells (*TRGV9, TRDV2*) (**Figure 4C**). The TC2-XIST (TC2), TC4-FOSB(TC4) and TC8-BCL2 (TC8) clusters were mostly derived from ascites, while other clusters were mainly from PBMCs (**Figure 4B**). Notably, TC2 and TC4 clusters were characterized by low expression of T cell markers (**Figures 4C, D**), such as *RORC*, *TRDC* and *ZBTB16* (37).

Since γδT cells are characterized by negative expression of *CD4* and *CD8* (38), we annotated TC4 as Vδ2 γδT cells and TC2 as non-Vδ2 γδT cells using R package *SingleR* (**Figure 4E**), suggesting that TC2 might represent a new subcluster of T cells. Furthermore, gene set enrichment analysis of the TC2 cluster revealed significant enrichment in genes of chromatin organization regulation, thus implicating its potential roles in shaping the immune community of T cells in ascites (**Figure 4F**). Interestingly, significantly enriched pathways in the TC2 cluster included the apoptosis, RIG-I-like receptor signaling, lysine degradation, and sulfur metabolism pathways, while the ribosome and oxidative phosphorylation (OXPHOS) pathways displayed low level enrichment (**Figure 4G**). Given that the OXPHOS pathway is a characteristic metabolic phenotype of T cells within the TIME (39), its low level enrichment, along with high enrichment of the apoptosis pathway and low enrichment of the ribosome function pathway, indicate a weakened immune function in TC2.

### Chemotherapy induced senescence and TCR clonal expansion of T cells derived from PBMC

Immunohistochemistry (IHC) results showed that CD8^+^ T cells were more abundant in recurrent tissue than primary lesions (**Supplementary Figure S4A**), suggesting that local CD8^+^T cells infiltration in tumor tissue is dynamic during progression of HGSOC. To investigate the peripheral T cell status, which may reflect the systemic immune response (40), we analyzed T cells from PBMCs before and after chemotherapy. Immune cells were classified into populations of myeloid cells, T cells, NK cells and B cells based on known markers (**Supplementary Figures S4B–E**), among which T cells were the most abundant population of immune cells (**Supplementary Figure S4D**). We also classified T/B cells according to the TCR/BCR distribution (**Supplementary Figure S4E**). Consistent with our scRNA-seq results (**Supplementary Figure S4F**), an increase of NK cells proportion after chemotherapy was detected by flow cytometry (**Supplementary Figures S6B, C**).

**Figure 5:**
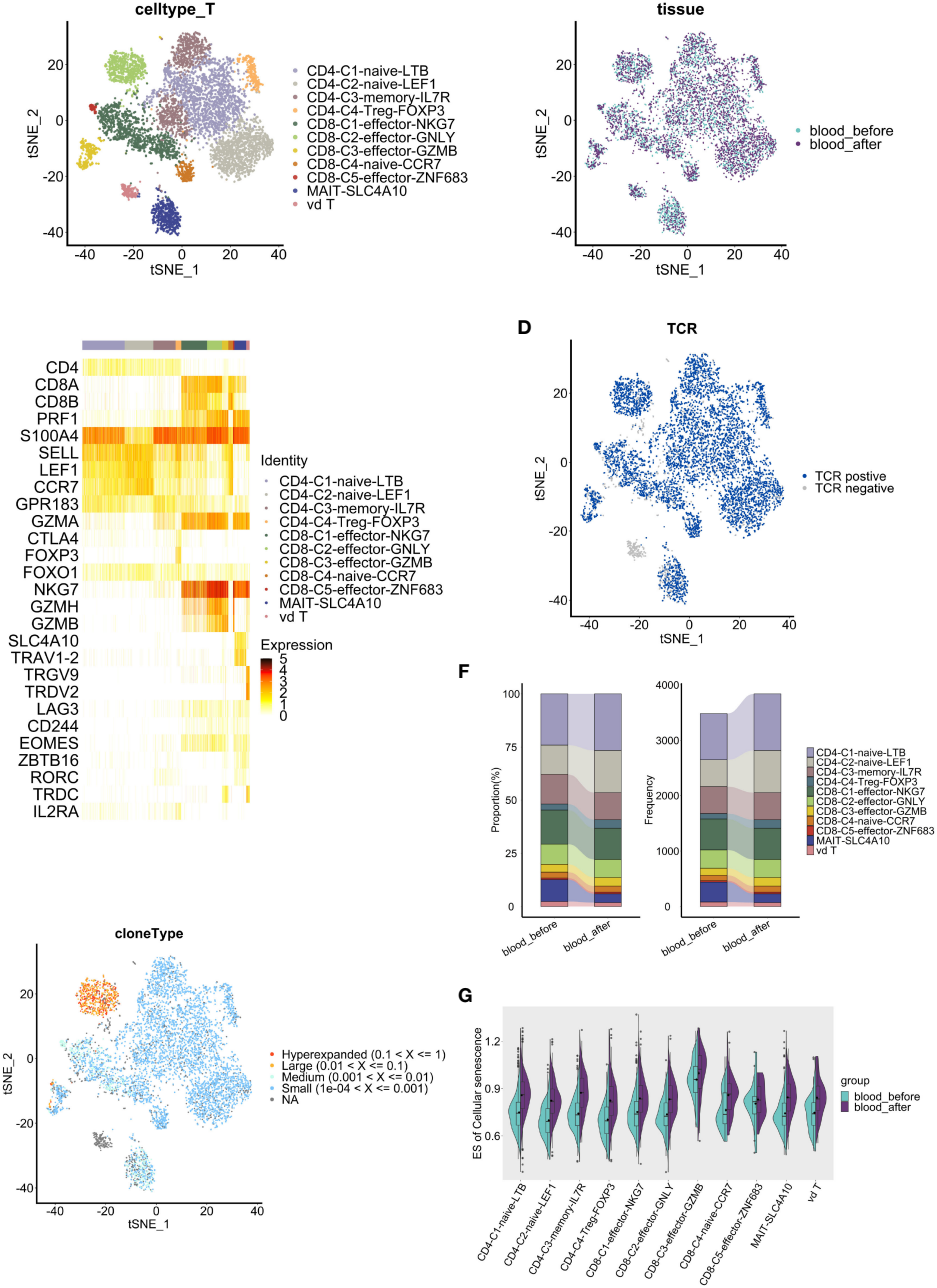
Comparative analysis of T cell features and dynamics in peripheral blood. **(A, B)** t-SNE visualization of T cells, colored by subclusters **(A)** and samples **(B)**. **(C)** Heatmap shows the expression level of marker genes in each T cell subcluster. **(D)** t-SNE visualization of TCRs identified in T cells. **(E)** t-SNE visualization of clonal expansion detected in T cells. **(F)** Proportion(left panel) and frequency(right panel) of T cell subclusters (y axis) in two blood samples(x axis). **(G)** Split violin plots show the enrichment level of cell senescence grouped by T subclusters and colored by samples. The results above are generated by comparison between samples (*blood_before, blood_after*).

In peripheral blood-derived T cells, we identified eleven subsets based on canonical markers (**Figures 5A–C**). The CD4^+^ cells included memory CD4^+^ T cells (*S100A4^+^GPR183^+^*), regulatory CD4^+^ T cells (T_reg_) (*FOXP3^+^IL2RA^+^*) and naïve CD4^+^ T cells (*CCR7^+^SELL^+^*) (constituted of CD4-C1-naïve-LTB and CD4-C2-naïve-LEF1). Five subsets of CD8^+^ T cells, including a naïve CD8^+^ T cell subset (*CCR7^+^ SELL^+^*) and four effector CD8^+^ T cell subsets (constituted of CD8-C1-effector-NKG7, CD8-C2-effector-GNLY, CD8-C3-effector-GZMB and CD8-C5-effector-ZNF683), expressed high levels of *GZMA* and *NKG7*. In addition, a MAIT subset (*SLC4A10^+^TRAV1-2^+^*) and a γδT subset (*TRGV9^+^TRDV2^+^*) were defined. Expression of exhaustion markers *LAG3*, *CD244* and *EOMES* were detected in all CD8^+^ T cell clusters (**Figure 5C**), among which the CD8^+^-C2-effector-GNLY group harbored the most extensive TCR clonal expansion (**Figures 5D, E**).

We further conducted cellular proportion analysis before and after chemotherapy. Among CD8^+^ T cells, C3-effector-GZMB (4.1% vs 3.7%) and C5-effector-ZNF683 (0.8% vs 0.7%) populations increased while C1-effector-NKG7 (14.7% vs 16.0%) and C2-effector-GNLY (8.4% vs 9.4%) populations decreased after chemotherapy (**Figure 5F**). Importantly, CD8^+^ GZMB T cells and CD8^+^ ZNF683 T cells are thought to be exhausted or exhausted-like cells, despite their ascribed cytotoxic function (10, 41). Therefore, changes of cellular proportion in CD8^+^ T cell subsets indicate the tendency towards an exhaust state, which may reflect the cumulative effects of chemotherapy.

To investigate whether chemotherapy affects the activation or exhaustion status of T cells *via* regulation of co-stimulatory molecules, which play important roles in the T cell response to antigenic stimuli (42), we compared the expression of co-stimulatory molecule receptors in PBMC-derived T cell clusters before and after chemotherapy. Low expression of immune checkpoint *PD-1* (*PLDCD1*) and *CTLA4* were observed (**Supplementary Figure S5A**), suggesting poor immune checkpoint blockade status, which is consistent with the low sensitivity of ovarian cancer to immune checkpoint therapy (43, 44). Among the co-stimulatory molecules, upregulation of *CD27*, which participates in the generation of memory CD8^+^ T cells (42), was observed in all CD4^+^ T cell clusters except for the CD4-C4-Treg-FOXP3 cluster; *TNFRSF14*, which enhances the tumor-specific immune response (45), increased in all CD4^+^ T cell clusters except for the CD4-C2-naive-LEF1 cluster; and *LAG3*, a marker of exhaustion (46), showed no significant change in CD4^+^ T cell clusters (**Supplementary Figures S5B–E**). We noted that most CD4^+^ T cell clusters generally showed a higher secretion of pro-inflammatorymolecules (*CD27, TNFRSF14*) after chemotherapy, indicating an activated state, while CD8^+^ T cell clusters did not show the same pattern. Among CD8^+^ T cells, higher expression of *CD27* was only observed specifically in the CD8-C2-effector-GNLY cluster, while elevated expression of *TNFRSF14* was observed in the CD8-C1-effector-NKG7 and CD8-C2-effector-GNLY clusters. Of note, we found a significant higher expression of exhaustion marker *LAG3* in most CD8^+^ effector T cell and γδT cell clusters (**Supplementary Figures S5F–J**), suggesting that impaired CD8^+^ effector T cells, which were promoted towards a more exhausted state by chemotherapy, are likely to have contributed to recurrence and a shorter PFS in this patient.

Next, to investigate whether chemotherapy can promote T cell senescence, we employed the GSVA method to compare the enrichment level of cellular senescence gene sets (obtained from KEGG, hsa04218) among PBMC-derived T cell clusters. T cell senescence is characterized by the accumulation of dysfunctional and terminally-differentiated cells (47), and a senescence-related gene set was significantly enriched in the CD8-C3-Effector-GZMB T cell cluster (**Figure 5G**). Of note, all T cell clusters, including the CD8-C3-Effector-GZMB cluster, gained a higher enrichment of cellular senescence-related genes after chemotherapy, strongly implying that chemotherapy promotes and accelerates T cell senescence (**Figure 5G**). Therefore, our results suggest that chemotherapy induces senescence-like T cell including CD8-C3-Effector-GZMB T cells, which may serve as a dysfunctional subpopulation with exhausted phenotype in HGSOC (10).

To prove our findings, flow cytometry and cytokines assay were performed (**Supplementary Figure S6A**). The levels of interleukin-6 (IL6), a classical senescence-associated secretory phenotype (SASP) and pro-inflammatory factor, were increased during the treatment period, while TNF-α and IFN-γ displayed a declined level (**Supplementary Figure S6E**). IL6/IL10 ratio increased gradually (**Supplementary Figure S6F**), implying a pro-inflammatory status in circulating immune system. However, despite once elevated, the proportion of CD8^+^ T effector cells (CD3^+^CD8^+^ CD25^+^) decreased after chemotherapy (**Supplementary Figures S6B, C**). Similarly, CD8^+^ T effector cells/T_reg_ cells (CD4 ^+^CD25 ^+^CD127^-^) ratio increased initially and then decreased after the treatment finished (**Supplementary Figure S6D**), suggesting a weakened antitumor activity. These results collectively indicate an initially activated but eventually suppressed phenotype of peripheral T cells after chemotherapy, probably caused by T cell senescence.

**Figure 6:**
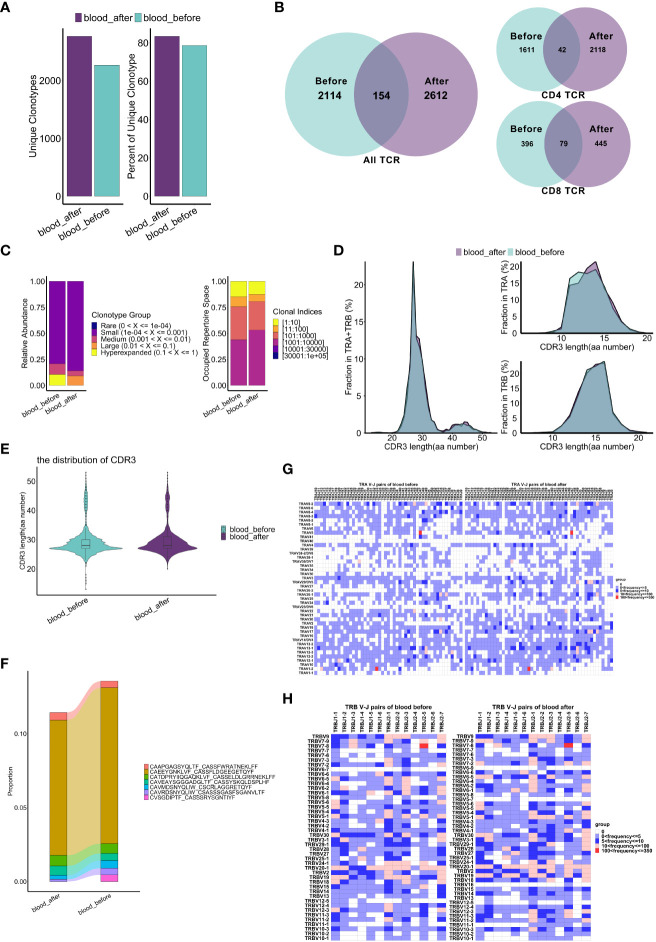
Comparative analysis of TCRs pre and post chemotherapy in peripheral blood. **(A)** Bar graphs show quantity and percentage of unique clonotypes. **(B)** Venn diagram showing the common and specific TCR of T cells (whole T cells, CD4^+^ T cells and CD8^+^ T cells). **(C)** Clonal homeostatic space representations (clonal space occupied by clonotypes of specific proportions) (left panel) and the relative proportional space occupied by specific clonotypes (right panel) of TCRs across samples. **(D)** Curve graphs show CDR3 aa length distribution of TCRs (TRA: α chains, TRB: β chains, aa: amino acid). **(E)** Violin plots show the CDR3 aa length distribution of TCR. **(F)** Dynamics of dominant CDR3 sequences of TCRs across samples pre and post chemotherapy, colored by the types of dominant sequences. **(G, H)** Heatmaps show frequency of V-J pairs in α chains **(G)** and β chains **(H)** among two samples. The results above are generated by comparison between samples (*blood_before, blood_after*).

Whether chemotherapy induces changes in TCR clonal expansion remains unclear. Therefore, we analyzed the dynamic of TCR repertoire during chemotherapy. Notably, we observed that the quantity and proportion of unique T cell clonotypes, which accounted for more than 70% of all clonotypes, increased after chemotherapy (**Figure 6A**). Only 154 unique clonotypes were shared before and after chemotherapy (**Figure 6B**). Similar trends were observed among CD4^+^ and CD8^+^ T cell subsets (**Supplementary Figures S7A, C**). These data strongly indicate that TCR clonal expansion was changed by chemotherapy. Interestingly, higher diversity indices (Shannon, Simpson, Chao and ACE index) were observed in CD4^+^ T cells compared to CD8^+^ T cells. The clonal overlap within CD4^+^ T cell clusters was not apparent while a strong overlap within CD8^+^ T cell clusters exists, especially between CD8-C1-effector-NKG7 and CD8-C2-effector-GNLY cells (**Supplementary Figures S7E, F**). Moreover, the relative abundance of highly expanded clonotypes decreased, and the low clonal index clonotypes occupied more repertoire space after chemotherapy (**Figure 6C** and **Supplementary Figures S7B, D**), suggesting that the TCR clonal expansion change may be explained by clonotypes with low clonal indices. Therefore, chemotherapy appears to have induced TCR clonal expansion in all T cells, and the influence on CD4^+^ TCR was more apparent.

As the complementarity determining region3 (CDR3) is the TCR region that directly contacts the antigen, thus playing a significant role in the interaction between the TCR and peptide-MHC complex (48), we next investigated whether chemotherapy changed the distribution of CDR3 within the α/β chains in different clonotypes. The distribution of amino acid (aa) length in the CDR3 α/β chain was mostly consistent, with 27aa comprising the most frequent length, both before and after chemotherapy (**Figures 6D, E**). Notably, the proportion of the CDR3 region with the same length slightly changed in CD4^+^ T cells (**Supplementary Figures S7G, H**) but remained almost unchanged in CD8^+^ T cells (**Supplementary Figures S7J, K**) after chemotherapy. Furthermore, clonotypes of dominant CDR3 sequences were reduced, and the CVSGDIPTF_CASSSRYSGNTIYF sequence disappeared after chemotherapy (**Figure 6F**). In CD4^+^ T cells, the clonotypes with a proportion of dominant sequences decreased significantly after chemotherapy (**Supplementary Figure S7I**), while the clonotypes in CD8^+^T cells remained almost unchanged, with the percentage of several dominant clonotypes increased slightly (**Supplementary Figure S7L**). These results suggest that chemotherapy changes TCR clonal expansion, while the influence on CD8^+^ T cells is not as apparent as on CD4^+^ T cells.

V(D)J rearrangement is the basis of TCR/BCR diversity, enabling immune responses of T/B cells to numerous antigens (16). Therefore, we further analyzed the bias of V-J pairs in alpha and beta chains before and after chemotherapy. Interestingly, TRAV5-TRAJ47, TRAV1-2-TRAJ33 and TRAV17-TRAJ54, the three most highly used V-J pairs of alpha chains, remained unchanged while other less-used pairs were changed much more after chemotherapy (**Figure 6G**). Among the beta chains, TRBV7-8-TRBJ2-5, TRBV20-1-TRBJ2-7 and TRBV20-1-TRBJ2-1 were the three most used V-J pairs before and after chemotherapy, while other less-used pairs were significant changed (**Figure 6H**). Furthermore, usage bias of V/J genes in T cell clonotypes was observed after chemotherapy (**Supplementary Figure S7M**). Collectively, based on clonotype and CDR3 analyses, these findings suggest that the TCR repertoire changes may be related to low-expanded clonotypes with low-frequency V-J pairs.

### Chemotherapy induces B cell activation and changes BCR clonal expansion

**Figure 7:**
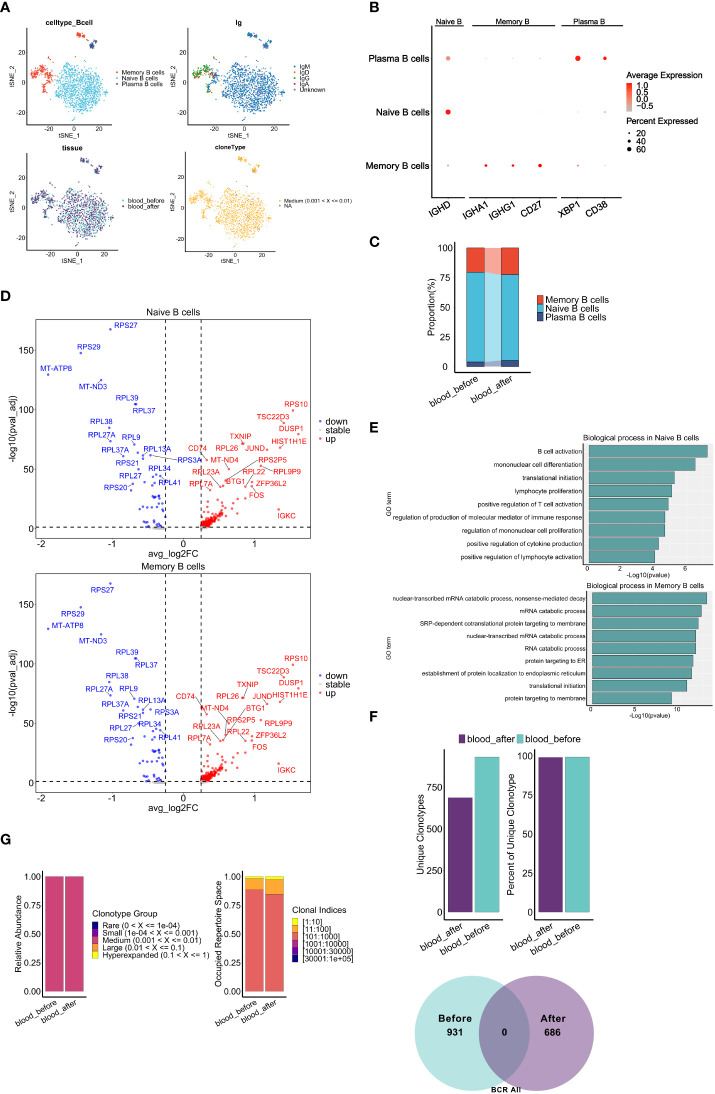
Characteristics of B cell subclusters before and after chemotherapy. **(A)** t-SNE visualization of B cells colored by cell types (top left), BCR isotypes (top right), derived-samples (bottom left) and clonal expansion status(bottom right). **(B)** Dot plots show expression level of marker genes of B cell types. **(C)** Proportion of B cell subclusters (y axis) in two blood samples(x axis). **(D)** Volcano plots show DEGs of naive B cells (top panel) and memory B cells (bottom panel) after chemotherapy compared with those before chemotherapy. **(E)** Gene set enrichment analysis of naive B cells (top panel) and memory B cells (bottom panel) after chemotherapy. The analyses were based on the Msigdbr GO database. **(F)** Bar graphs top panel and venn diagram bottom panel show the change of frequency and fraction of unique clonotypes, colored by collection time. **(G)** Clonal homeostatic space representations (clonal space occupied by clonotypes of specific proportions) (left panel) and the relative proportional space occupied by specific clonotypes (right panel) of BCR across samples pre and post chemotherapy. The results above are generated by comparison between samples (*blood_before, blood_after*).

Recently, different subsets of B cells have been reported to play important roles during the dynamic progression of tumors (49). For example, the ICOSL^+^ subset of B cells has been shown to emerge after chemotherapy and may enhance the immune response in breast cancer (50). Furthermore, IgA derived from tumors has been shown to antagonize the growth of OC by governing coordinated responses of tumor cells, T cells and B cells (51). To assess the influence of chemotherapy on peripheral B cell phenotype and function, we analyzed scRNA-seq and scBCR-seq data. A total of 1690 B cells were obtained, and 1631 cells with full-length productive paired IGH-IGK/IGL chains were retained for further analysis. Based on the expression of canonical markers, the B cells were categorized into three distinct subsets: naïve B cells (*IGHD*), memory B cells (*CD27, IGHA1, IGHG1*) and plasma cells (*CD38, XBP1*) (**Figures 7A, B**). Comprised of IgM, IgD, IgG and IgA isotypes, naïve B cells accounted for majority of peripheral B cells. All B cells were median-expanded (**Figure 7A**). The percentages of plasma cells (5.10% vs 3.92%) and memory B cells (22.45% vs 20.54%) increased, and the percentage of naïve B cells decreased (72.45% vs 75.54%) after chemotherapy (**Figure 7C**), suggesting that neoantigens induced by chemotherapy may cause naïve B cells to differentiate into plasma or memory B cells. Several key genes related to NF-κB signaling(CD74), MAPK signaling(FOS, DUSP1) pathways, were markedly upregulated in both naïve and memory cells after chemotherapy, suggesting that chemotherapy may induce B cell activation, proliferation and maturation. (52, 53) (**Figure 7D**). Using R package Clusterprofiler we found that a variety of inflammatory response pathways were significantly enriched in naïve B cells after chemotherapy, while protein synthesis and RNA catabolism pathways were enriched in memory B cells (**Figure 7E**).

Next, we explored the dynamics of BCR repertoires during chemotherapy. Interestingly, we observed a consistent proportion of unique clonotypes before and after chemotherapy, and no unique clonotypes were shared (**Figure 7F**), suggesting significant changes in BCR clonal expansion may be primarily attributed to the chemotherapy. Of note, no apparent increase was observed in the relative abundance of clonotypes and the occupied space of corresponding clonal indices (**Figure 7G**), which defers from the results of TCR analysis (**Figure 6C**). In addition, there were no significant differences in the CDR3 length distribution, while the proportion of CDR3 with the same length was less after chemotherapy (**Supplementary Figures S8A, B**). Furthermore, a mild difference in distribution was observed in memory B cells but not naïve B cells (**Supplementary Figure S8D**). Notably, completely different CDR3 dominant sequences (**Supplementary Figure S8C**) and usage bias of the V-J gene segments in memory B cells relative to naïve B cells after chemotherapy (**Supplementary Figures S7E, F**) were observed. In summary, chemotherapy promoted peripheral B cell activation and changed clonal expansion of the BCR repertoire, potentially contributing to the response to neo-antigens induced by chemotherapy.

## Discussion

HGSOC is characterized by disseminated abdominal spread, easy of recurrence, and chemoresistance in advanced-stage patients. Malignant abdominal ascites provides a complex cancerous and immunological microenvironment for tumor progression and recurrence. Single-cell sequencing provides a vital method to better understand the fundamental mechanisms of cancer relapse and chemoresistance. In this study, we revealed the intratumor heterogeneity, immunosuppressive features in ascites, and dynamic changes of immune status of PBMC in a relapsed chemo-resistant HGSOC patient after chemotherapy. Furthermore, we demonstrated that chemotherapy remodel TIME in peripheral blood and change the clonal expansion of TCR/BCR. These findings highlight the impact of chemotherapy on TIME, which may contribute to future development of novel immune-modulatory strategy for relapsed chemo-resistant ovarian cancer patients.

We first investigated whether intrinsic properties of tumor cells contribute to chemoresistance. FTE markers (*PAX8, KRT7*) were highly expressed in all subclusters of epithelial cells, indicating that the tumor may originate from fallopian tube (8). Of note, EC3 subcluster showed high expression of chemoresistance related genes and was comprised of a large proportion of G2/M cells, along with an elevated metabolism level, which is associated with progression and platinum-based chemoresistance in HGSOC (54, 55). High heterogeneity and high proliferation ability of epithelial cells were probably caused by CNVs (56, 57). Compared with those in sensitive HGSOC samples, chemo-resistant recurrent epithelial cells showed higher CNVs level, implying that EOC_Tumor may be in a more malignant state. Since cancer somatic mutations can generate neoantigens (58), an obvious upregulation of antigen presentation genes across all cancer cell clusters suggests clonal expansion of TCR or BCR to neoantigens. Consistently, IFN-associated genes, were highly expressed in cancer cells from both GSE154600 and our case, which might predict better prognosis. However, a shortened PFS and platinum-free interval (PFI), along with an increased frequency of chemotherapy of this patient still needs more investigation.

Then we further investigate whether status of TIME contribute to chemo-resistance of HGSOC. Previous study has shown that the high expression of M2 marker in macrophages is associated with poor prognosis of ovarian cancer (59), and upregulated M2 marker is considered to imply immune-suppressive phenotype (60). Our patients showed high expression of M2 signatures in both tumor-infiltrated and ascites-resident macrophages, indicating that M2 TAMs polarization may promote chemo-resistance. Our findings also suggest that peripheral monocyte/macrophage subsets may migrate to the ascites or tumors and be educated to perform different functions in the TIME. Integrating GSE154600 and our data, we affirmed our findings that chemo-resistant tumors may share signatures of immunosuppressive myeloid phenotype. In addition, the predominant co-expression of *GPNMB* in myeloid cells (**Supplementary Figure S3I**) and CD44 in cancer cells (**Supplementary Figure S1C**) in chemo-resistant samples may provide us with the mechanism underlying chemo-resistance. Macrophages-secreted GPNMB induces cancer stemness *via* CD44 on cancer cells (61), suggesting that enhanced cancer cell stemness may explain the shorter PFS of this patient, despite high expression of IFN and antigen presentation-related genes. Given that cancer cell and TIME are cross-talked, dual target both parts simultaneously may overcome chemoresistance. The role of γδ T cells in tumor is still unclear and the residency of γδ T cells may play pro- or anti-tumorigenicity (42). Besides, low-activated and immunosuppressive ascites-derived γδ T cells were observed in epithelial ovarian cancer (62), and low metabolism level of T cells can lead to antitumor dysfunction (63). Similarly, we found that ascites-derived γδ T cells had decreased metabolic pathways and increased apoptosis pathways, indicating its immunosuppressive status. These observations suggest that immunosuppressive TME may play an essential role in chemo-resistant HGSOC.

So far, the impact of chemotherapy on phenotype and function of peripheral T/B cells in HGSOC still requires elucidation. Our findings revealed that that chemotherapy promote the transformation of T cells to an exhaustive and dysfunctional status, which interact with enriched M2-like TAM to lead to immune dysfunction, as previous reported (60). In addition, our data showed that chemotherapy leads to T cell senescence, in line with increased IL-6 in peripheral blood, which are hallmarks of cellular senescence (**Supplementary Figure S6**) (64). Since senescent T cells compose suppressive TME (65), our findings indicate chemotherapy induced immune-suppressive transformation in peripheral blood circulation. Furthermore, our research on TCR reveals a clonal expansion and V(D)J rearrangement, which is not exactly consistent with other study which found that overall repertoire diversity remains stable after the chemotherapy (66). Besides, our results also indicates that chemotherapy leads to the activation, proliferation and maturation of peripheral B cells, suggesting that chemotherapy-induced neoantigens may play a pivotal role in anti-tumor response of B cells through collaboration with T cells (67).

The limitations of this study should be noted here. First, lack of large-number paired clinical resources of relapsed chemo-resistant samples developed from chemo-sensitive, including tumor, ascites and PBMC, leads to inadequate clarification of our conclusion. Second, elucidating mechanism of chemoresistance in HGSOC requires *in vitro* and *in vivo* experiments.

In summary, through integrating cross-sectional analysis of single-cell RNA, TCR and BCR profiles from paired ascites, tumor and peripheral blood samples, we provided important insight into the TME in an HGSOC patient with several cycles of relapse and chemo-resistance. We revealed the variable changes in clonal expansion of the TCR and BCR, laying the foundation for understanding of host anti-tumor immune mechanisms and immune reconstruction induced by chemotherapy. Our research also provides an in-depth exploration of cancerous and immune environments of HGSOC with relapsed platinum-resistance, which may facilitate the development of novel chemotherapy in combination with anti-senescence agents to improve the prognosis and overall survival of ovarian cancer patients.

## Data availability statement

The dataset presented in this study is publicly accessible in the GEO database, accession number GSE213243.

## Ethics statement

The studies involving human participants were reviewed and approved by the Ethics Committee of Nanfang Hospital (NO. NFEC-2021-424). The patients/participants provided their written informed consent to participate in this study. Written informed consent was obtained from the individual(s) for the publication of any potentially identifiable images or data included in this article.

## Author contributions

Conceptualization, YN. Software: RL and JX. Methodology: YR, RL and YN. Formal analysis and statistics: RL, JX and LG. Investigation: YR, RL and SC. Resources: FM. Writing– original draft: YR, HF and RL. Writing– review & editing: YR, HF, RL, YN, YL and FM. Visualization: RL, YR, HF, JX and LG. Supervision: YN, YL, and FM. All authors contributed to the article and approved the submitted version.

## Funding

This work was supported by the National Key Research and Development Project of China (2019YFA0903800), the National Natural Science Foundation of China (81971903), the Guangdong Basic and Applied Basic Research Foundation (2021A1515010779), the Nanfang Hospital President’s Fund (2019B019) and the Innovative experiment program of college students of Guangdong Province, China (No. S202012121095, S202112121130, S202112121136, X202212121322).

## Acknowledgments

We thank LetPub (www.letpub.com) for linguistic assistance and pre-submission expert review.

## Conflict of interest

The authors declare that the research was conducted in the absence of any commercial or financial relationships that could be construed as a potential conflict of interest.

## Publisher’s note

All claims expressed in this article are solely those of the authors and do not necessarily represent those of their affiliated organizations, or those of the publisher, the editors and the reviewers. Any product that may be evaluated in this article, or claim that may be made by its manufacturer, is not guaranteed or endorsed by the publisher.

## Supplementary material

Supplementary Figure S1 Characteristics of tumor cells revealed at single-cell resolution. **(A)** Fraction and frequency of tumor cells (x axis) from samples (*Tumor, Ascite*) in each subcluster (y axis). **(B)** Heatmap displays the enriched pathways in tumor cell subclusters performed by GSVA analysis. **(C)** t-SNE plot displays main cell types from five HGSOC samples (*T59, T76, T77, T89, T90*). **(D)** Violin plots display expression of selected chemoresistance related genes in tumor cell populations of six samples (*Tumor, T59, T76, T77, T89, T90*). The distribution of the per cell signature expression was based on normalized data. **(E)** Heatmap displays large-scale CNVs of epithelial tumor cells compared to myeloid cells using inferCNV. The annotation on the right indicates the corresponding sample sources. The red represents CNV amplifications and blue represents CNV deletion. **(F)** Heatmap displays top 10 differentially-expressed genes (DEGs) of EC3 in each tumor cell subcluster. **(G)** Expression profiles of top 10 DEGs (shown in **Supplementary Figure 1F**) were examined by Spearman correlation coefficient between epithelial tumor cells in six HGSOC samples (*Tumor, T59, T76, T77, T89, T90*). **(H)** Violin plots show the enrichment level of interferon-associated signature genes (shown in **Figure 2H**) among each cell type. Distribution of the per cell signature expression was based on the GSVA scores. **(I)**Kaplan-Meier curve for TCGA-OV cohorts based on expression of interferon-associated signature genes (shown in **Figure 2H**). The groups are distinguished by median enrichment scores. P value is calculated with log-rank test.

Supplementary Figure S2 The landscape of immune cells in ascites, tumor and peripheral blood. (**A** and **B**) t-SNE plots display main cell types from three samples (Tumor, Ascite, blood_before) before chemotherapy, colored by immune cell clusters **(A)** and the origins **(B)**. **(C)** Dot plots display the expression level of signature genes in each immune cell cluster. **(D)** Frequency and fractions of each immune cell cluster among three samples (*Tumor, Ascite and blood_before*). (**E** and **F**) Heatmaps show selected markers(E) and DEGs (F) in each myeloid cell cluster.

Supplementary Figure S3 Characteristics of myeloid cells in distinct TMEs and chemokine expression in tumor cell subtypes. **(A, B)** Dot plots show expression level of chemokines ligand family CCL **(A)** and CXCL **(B)** in tumor cell subclusters. **(C, D)** Dot plots show expression level of chemokines receptors family CCR **(C)** and CXCR **(D)** in macrophage cell subclusters. **(E, F)** Ligand-receptor interactions from tumor cell subclusters to APOE+ Macrophages **(E)** and ADAP2+ Macrophages **(F)** in samples (*Tumor, Ascite*). P values are represented by the size of each circle. The color gradient shows the level of interaction. **(G)** Fraction of immune cells from six samples (*Tumor, T59, T76, T77, T89, T90*). **(H)** t-SNE plot displays myeloid cell types from six samples (*Tumor, T59, T76, T77, T89, T90*). **(I)** Boxplots of immune phenotype related gene changes (*CD163, MRC1, CD68, SOCS3, TREM2, GPNMB,* respectively) across macrophage cells from six samples (*indicates a p value < 0.01, ** indicates a p value < 0.001, *** indicates a p value < 0.0001, NS indicates no significance)..

Supplementary Figure S4 Landscape of immune cells in peripheral blood collected pre and post the fourth course of chemotherapy. **(A)** Representative H&E and CD8 IHC images for primary (pr_T) and relapsed (re_T) tumor regions shown at ×4 magnification, scale bar 600 μm; ×10 magnification, scale bar 300 μm; and ×20 magnification, scale bar 200 μm. **(B)** t-SNE visualization of immune cell clusters from samples (*blood_before, blood_after*). **(C)** Heatmap shows the expression level of marker genes in myeloid cells, T cells, NK cells and B cells. **(D)** Fraction and frequency of immune cells (x axis) from samples (*blood_before, blood_after*) in each cell type (y axis). **(E)** t-SNE visualization of TCR (top panel) and BCR (bottom panel) distrubtion in all immune cells. **(F)** Proportion of immune cells among samples (*blood_before, blood_after*).

Supplementary Figure S5 Comparative analysis of co-stimulatory molecules on T cell clusters in peripheral blood. **(A)** t-SNE visualization of the expression level of *CTLA4* and *PDCD1* in T cells from PBMCs. **(B–E)** Boxplots show the expression level of *CD27, TNFRSF14, TNFRSF1A* and *LAG3* in each CD4^+^ T cell cluster pre and post chemotherapy. **(F–I)** Boxplots show the expression level of *CD27, TNFRSF14, TNFRSF1A* and *LAG3* in each CD8^+^ T cell cluster pre and post chemotherapy. **(J)** Boxplots show the expression of *CD27, TNFRSF14, TNFRSF1A* and *LAG3* in γδ T cell cluster pre and post chemotherapy. The results above are generated by comparison between samples (*blood_before, blood_after*).

Supplementary Figure S6 Immune function assay of peripheral blood during the treatment of chemotherapy. **(A)** Workflow of the flowcytometry assessing peripheral immune cells and cytokine assay in peripheral blood. Sorting standard of immune cell populations are shown. **(B, C)** Representative flow cytometry plots **(B)** and line charts **(C)** of the proportion of NK cells, CD8^+^ T_eff_ and T_reg_ in peripheral blood during the treatment of chemotherapy. **(D–F)** Line charts display the change of CD8^+^ T_eff_/T_reg_ ratio **(D)**, cytokines concentration **(E)** and IL-6/IL-10 ratio **(F)**. (T1: Before the second chemotherapy began; T2: Two days after the sixth chemotherapy; T3: Fourteen days after the sixth chemotherapy when the sample blood_after was sequenced).

Supplementary Figure S7 Comparative analysis of TCRs in CD4^+^ and CD8^+^ T cells across samples pre and post chemotherapy. (**A**, **C**) Quantity and percentage of unique clonotypes for CD4^+^ T cells **(A)** and CD8^+^ T cells **(C)** between samples pre and post chemotherapy. (**B**, **D**) Clonal homeostasis and clonal proportion of CD4^+^ T cells (**B**) and CD8^+^ T cells **(D)** between samples pre and post chemotherapy. (**E**, **F**) Clonotypes diversity measures based on subclusters (left panel) using Shannon, Inverse Simpson, Chao and ACE index. Clonotypes overlap quantifications by clusters (right panel) in CD4^+^ T cells **(E)** and CD8^+^ T cells **(F)**. (**G**, **J**) Curve graphs show TCR CDR3 aa length distribution of TRA (upper right) and TRB (bottom right) and both(left) in CD4^+^ T cells **(G)** and CD8^+^ T cells **(J)** across samples pre and post chemotherapy. (TRA: α chains, TRB: β chains, aa: amino acid). (**H**, **K**) Violin plots show CDR3 aa length distribution in CD4^+^ T cells **(H)** and CD8^+^ T cells **(K)**. (**I**, **L**) Dynamics of dominant CDR3 sequences of TCRs in CD4^+^ T cells **(I)** and CD8^+^ T cells **(L)**. **(M)** Bar graphs show the fraction of V and J genes in α chains and β chains among T cells. Genes with significant changes are labeled red. (*indicates a FDR < 0.01, ** indicates a FDR < 0.001, *** indicates a FDR < 0.0001, **** indicates a FDR < 0.00001). The results above are generated by comparison between samples (*blood_before, blood_after*).

Supplementary Figure S8 Comparative analysis of BCRs in naïve and memory B cells across samples pre and post chemotherapy. **(A)** Curve graphs show CDR3 aa length distribution of IGL (upper right), IGH (bottom right) and both (left) of all BCRs across samples pre and post chemotherapy. **(B)** Violin plots show the CDR3 aa length distribution of IGH chains plus IGL chain. **(C)** Dynamics of dominant CDR3 sequences of BCRs across samples pre and post chemotherapy, colored by types of dominant sequences. **(D)** Violin plots show distributions of CDR3 length of naïve B cells (left) and memory B cells(right) across samples pre and post chemotherapy. (**E** and **F**) Bar graphs show the fraction of immunoglobulin IGHV (upper left), IGHJ (upper right), IGLV/IGKV (bottom left), and IGLJ/IGKJ (bottom right) genes in naïve B cells **(E)** and memory B cells **(F)**. Genes with significant changes are labeled red. (*indicates a FDR < 0.01, ** indicates a FDR < 0.001, *** indicates a FDR < 0.0001, **** indicates a FDR < 0.00001). The results above are generated by comparison between samples (*blood_before, blood_after*).
