# Supplementary figures and images for: Single-cell sequencing reveals effects of chemotherapy on the immune landscape and TCR/BCR clonal expansion in a relapsed ovarian cancer patient
Source: Front Immunol. 2022 Sep 28;13:985187. doi: 10.3389/fimmu.2022.985187 (PMC9555851; doi:10.3389/fimmu.2022.985187)

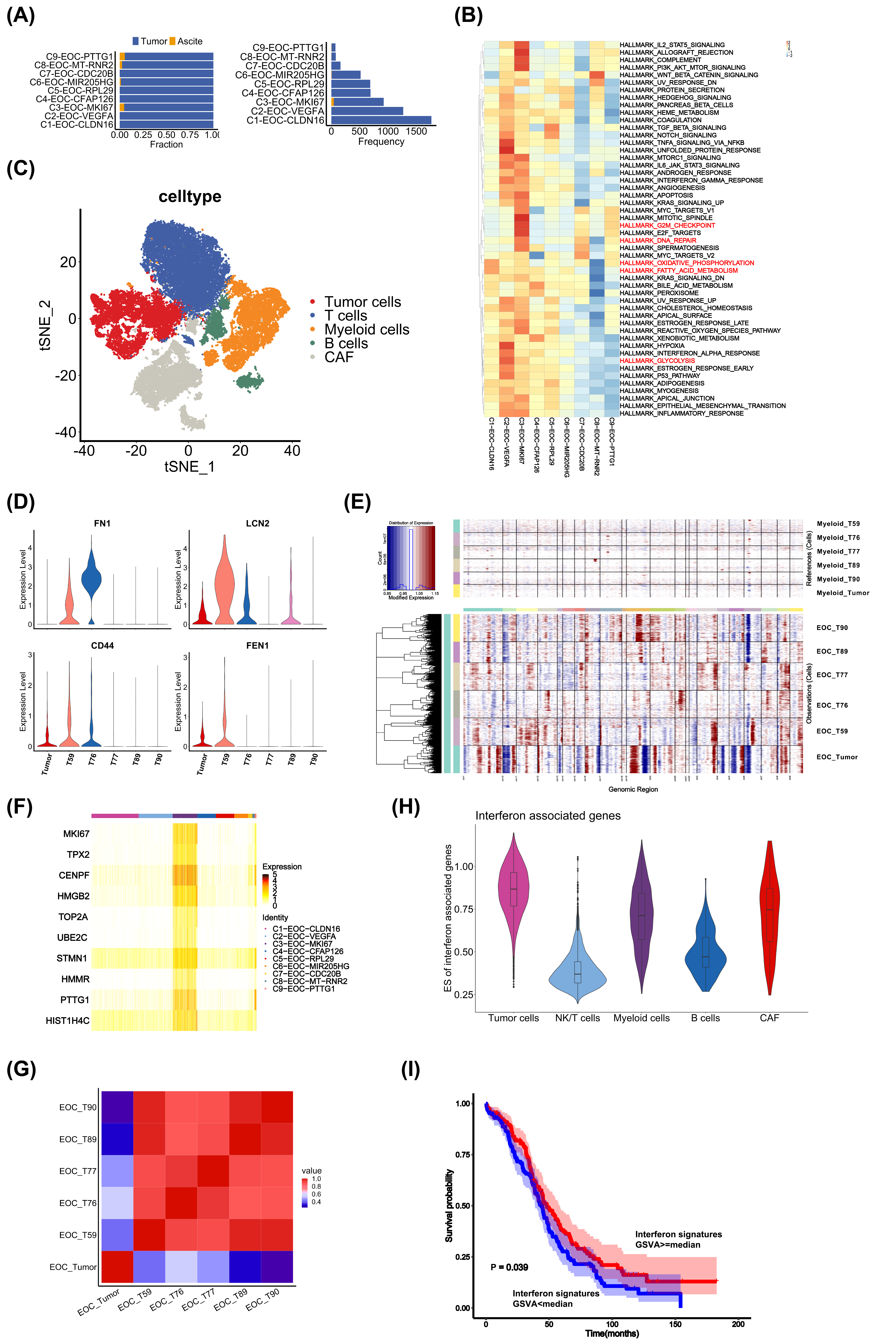

Supplement: Supplementary Figure S1 — Characteristics of tumor cells revealed at single-cell resolution. (A) Fraction and frequency of tumor cells (x axis) from samples (Tumor, Ascite) in each subcluster (y axis). (B) Heatmap displays the enriched pathways in tumor cell subclusters performed by GSVA analysis. (C) t-SNE plot displays main cell types from five HGSOC samples (T59, T76, T77, T89, T90). (D) Violin plots display expression of selected chemoresistance related genes in tumor cell populations of six samples (Tumor, T59, T76, T77, T89, T90). The distribution of the per cell signature expression was based on normalized data. (E) Heatmap displays large-scale CNVs of epithelial tumor cells compared to myeloid cells using inferCNV. The annotation on the right indicates the corresponding sample sources. The red represents CNV amplifications and blue represents CNV deletion. (F) Heatmap displays top 10 differentially-expressed genes (DEGs) of EC3 in each tumor cell subcluster. (G) Expression profiles of top 10 DEGs (shown in Supplementary Figure 1F ) were examined by Spearman correlation coefficient between epithelial tumor cells in six HGSOC samples (Tumor, T59, T76, T77, T89, T90). (H) Violin plots show the enrichment level of interferon-associated signature genes (shown in Figure 2H ) among each cell type. Distribution of the per cell signature expression was based on the GSVA scores. (I)Kaplan-Meier curve for TCGA-OV cohorts based on expression of interferon-associated signature genes (shown in Figure 2H ). The groups are distinguished by median enrichment scores. P value is calculated with log-rank test. [file Image_1.tif]

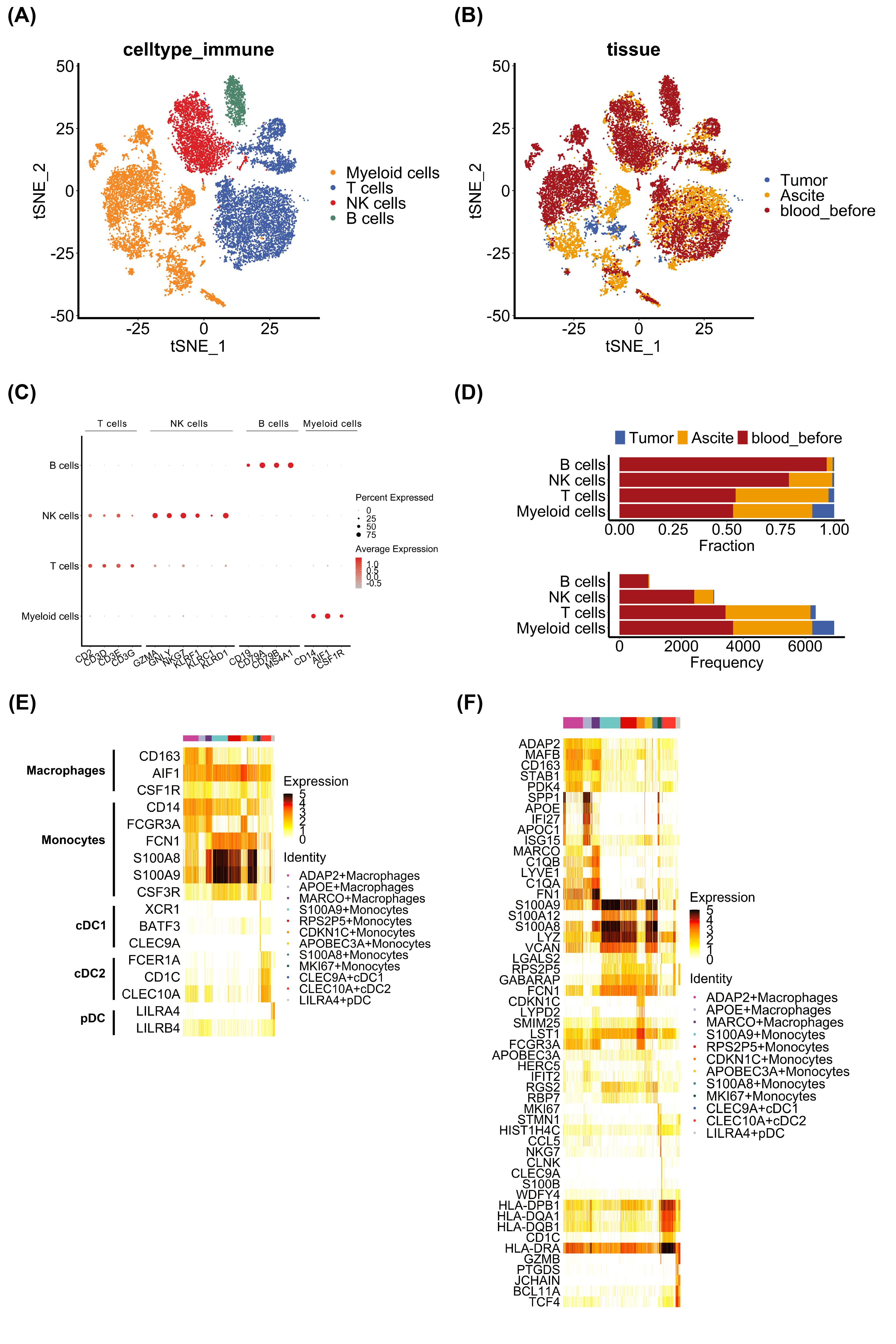

Supplement: Supplementary Figure S2 — The landscape of immune cells in ascites, tumor and peripheral blood. (A and B) t-SNE plots display main cell types from three samples (Tumor, Ascite, blood_before) before chemotherapy, colored by immune cell clusters (A) and the origins (B). (C) Dot plots display the expression level of signature genes in each immune cell cluster. (D) Frequency and fractions of each immune cell cluster among three samples (Tumor, Ascite and blood_before). (E and F) Heatmaps show selected markers(E) and DEGs (F) in each myeloid cell cluster. [file Image_2.tif]

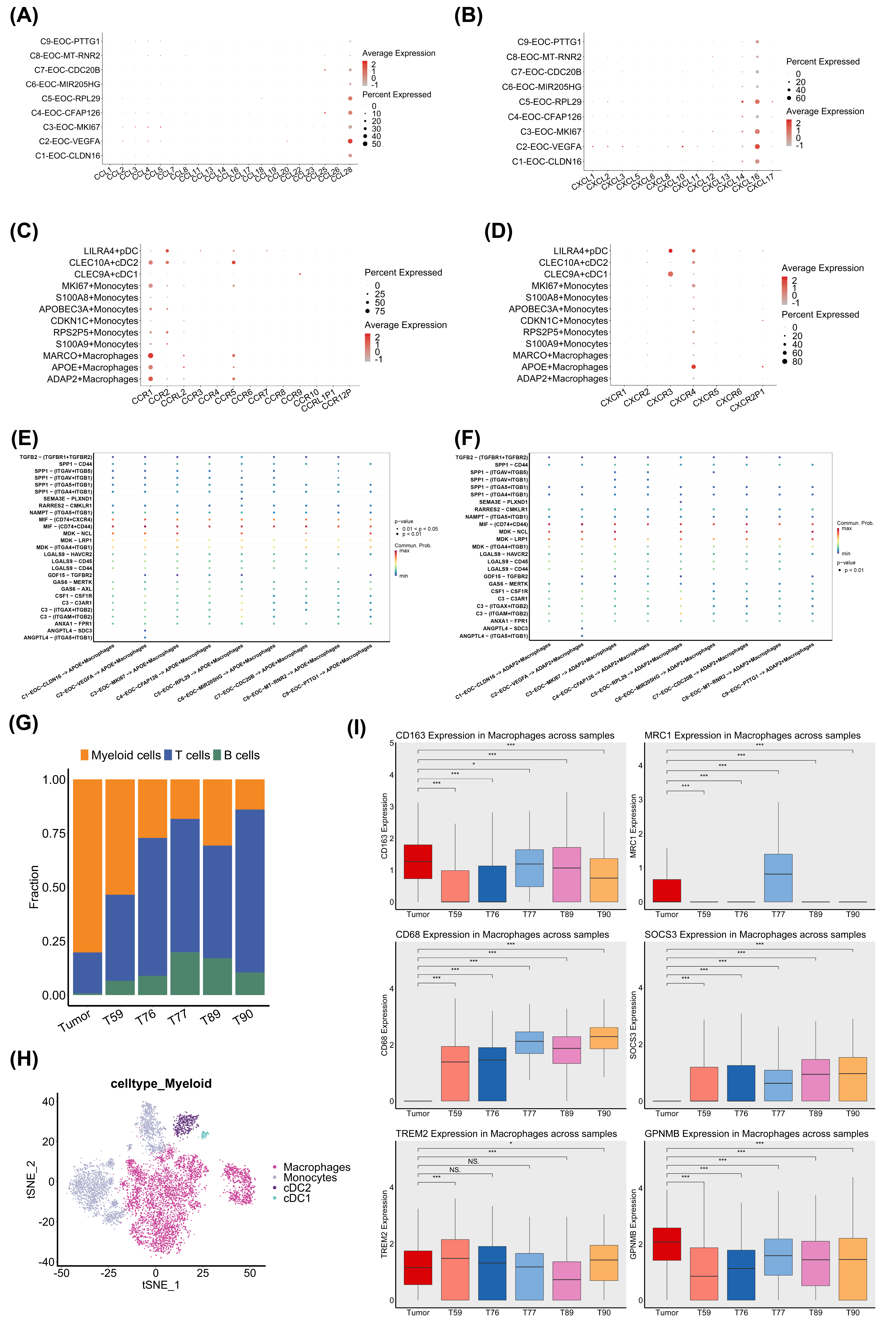

Supplement: Supplementary Figure S3 — Characteristics of myeloid cells in distinct TMEs and chemokine expression in tumor cell subtypes. (A, B) Dot plots show expression level of chemokines ligand family CCL (A) and CXCL (B) in tumor cell subclusters. (C, D) Dot plots show expression level of chemokines receptors family CCR (C) and CXCR (D) in macrophage cell subclusters. (E, F) Ligand-receptor interactions from tumor cell subclusters to APOE+ Macrophages (E) and ADAP2+ Macrophages (F) in samples (Tumor, Ascite). P values are represented by the size of each circle. The color gradient shows the level of interaction. (G) Fraction of immune cells from six samples (Tumor, T59, T76, T77, T89, T90). (H) t-SNE plot displays myeloid cell types from six samples (Tumor, T59, T76, T77, T89, T90). (I) Boxplots of immune phenotype related gene changes (CD163, MRC1, CD68, SOCS3, TREM2, GPNMB, respectively) across macrophage cells from six samples (*indicates a p value < 0.01, ** indicates a p value < 0.001, *** indicates a p value < 0.0001, NS indicates no significance).. [file Image_3.tif]

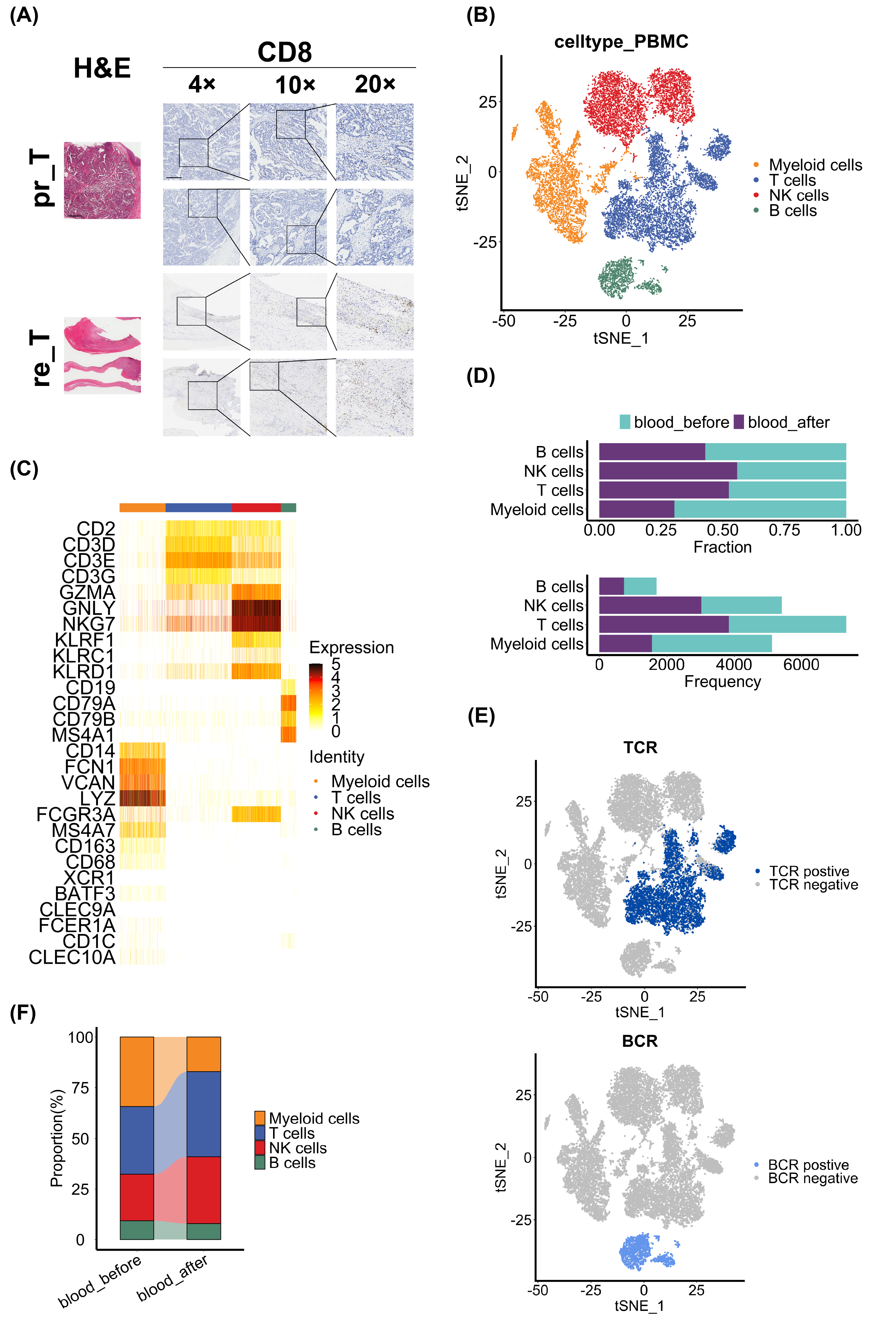

Supplement: Supplementary Figure S4 — Landscape of immune cells in peripheral blood collected pre and post the fourth course of chemotherapy. (A) Representative H&E and CD8 IHC images for primary (pr_T) and relapsed (re_T) tumor regions shown at ×4 magnification, scale bar 600 μm; ×10 magnification, scale bar 300 μm; and ×20 magnification, scale bar 200 μm. (B) t-SNE visualization of immune cell clusters from samples (blood_before, blood_after). (C) Heatmap shows the expression level of marker genes in myeloid cells, T cells, NK cells and B cells. (D) Fraction and frequency of immune cells (x axis) from samples (blood_before, blood_after) in each cell type (y axis). (E) t-SNE visualization of TCR (top panel) and BCR (bottom panel) distrubtion in all immune cells. (F) Proportion of immune cells among samples (blood_before, blood_after). [file Image_4.tif]

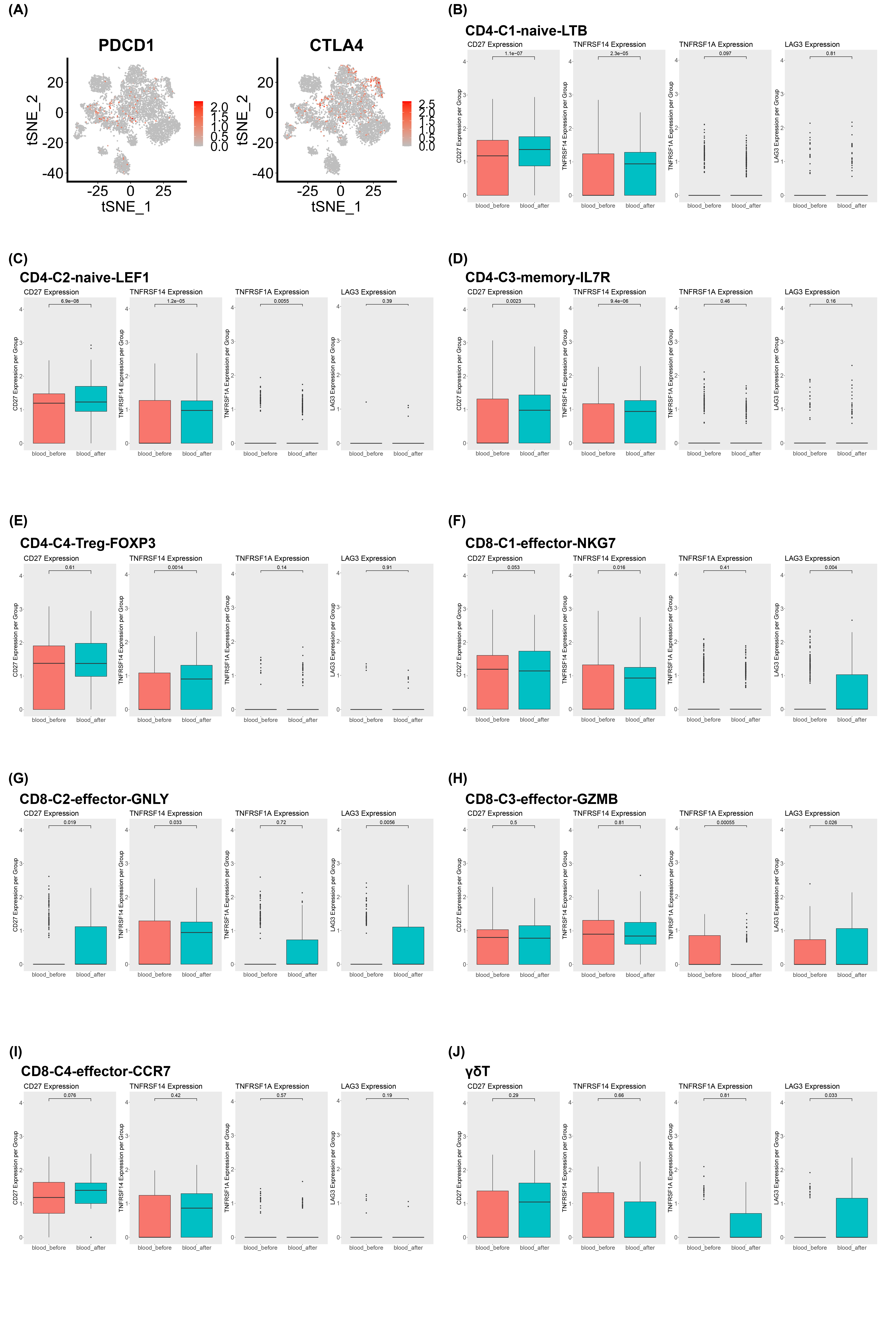

Supplement: Supplementary Figure S5 — Comparative analysis of co-stimulatory molecules on T cell clusters in peripheral blood. (A) t-SNE visualization of the expression level of CTLA4 and PDCD1 in T cells from PBMCs. (B–E) Boxplots show the expression level of CD27, TNFRSF14, TNFRSF1A and LAG3 in each CD4+ T cell cluster pre and post chemotherapy. (F–I) Boxplots show the expression level of CD27, TNFRSF14, TNFRSF1A and LAG3 in each CD8+ T cell cluster pre and post chemotherapy. (J) Boxplots show the expression of CD27, TNFRSF14, TNFRSF1A and LAG3 in γδ T cell cluster pre and post chemotherapy. The results above are generated by comparison between samples (blood_before, blood_after). [file Image_5.tif]

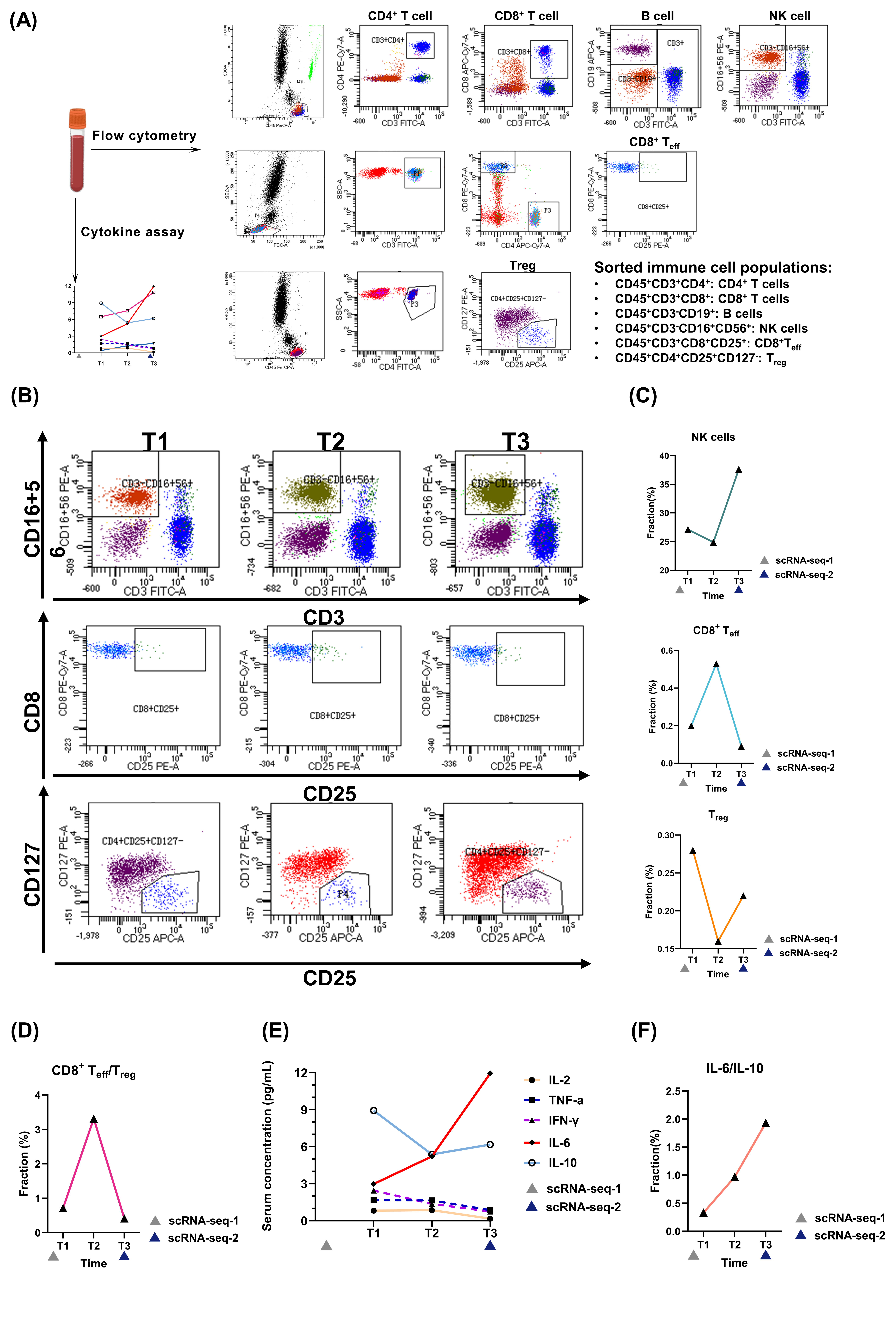

Supplement: Supplementary Figure S6 — Immune function assay of peripheral blood during the treatment of chemotherapy. (A) Workflow of the flowcytometry assessing peripheral immune cells and cytokine assay in peripheral blood. Sorting standard of immune cell populations are shown. (B, C) Representative flow cytometry plots (B) and line charts (C) of the proportion of NK cells, CD8+ Teff and Treg in peripheral blood during the treatment of chemotherapy. (D–F) Line charts display the change of CD8+ Teff/Treg ratio (D), cytokines concentration (E) and IL-6/IL-10 ratio (F). (T1: Before the second chemotherapy began; T2: Two days after the sixth chemotherapy; T3: Fourteen days after the sixth chemotherapy when the sample blood_after was sequenced). [file Image_6.tif]

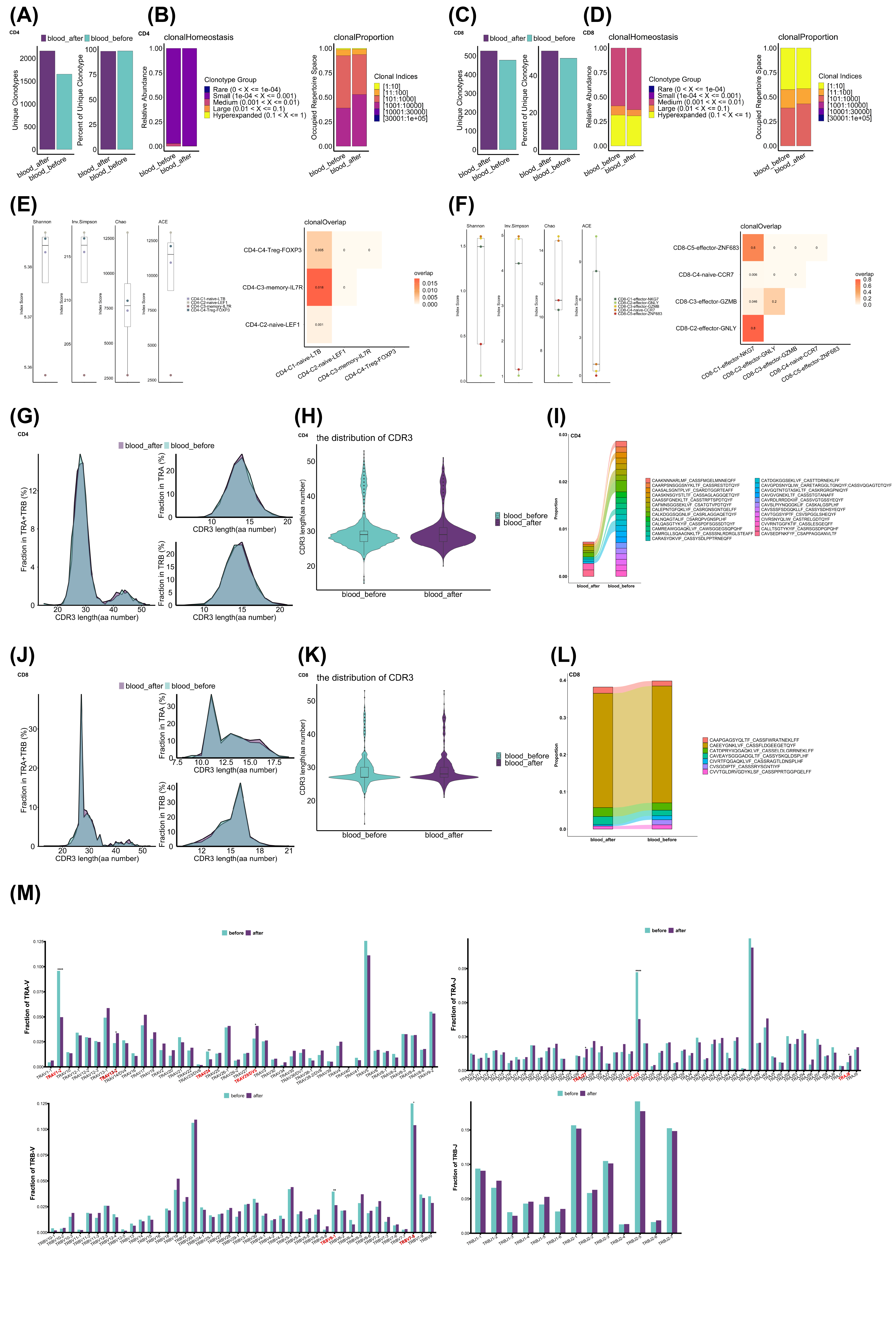

Supplement: Supplementary Figure S7 — Comparative analysis of TCRs in CD4+ and CD8+ T cells across samples pre and post chemotherapy. (A, C) Quantity and percentage of unique clonotypes for CD4+ T cells (A) and CD8+ T cells (C) between samples pre and post chemotherapy. (B, D) Clonal homeostasis and clonal proportion of CD4+ T cells (B) and CD8+ T cells (D) between samples pre and post chemotherapy. (E, F) Clonotypes diversity measures based on subclusters (left panel) using Shannon, Inverse Simpson, Chao and ACE index. Clonotypes overlap quantifications by clusters (right panel) in CD4+ T cells (E) and CD8+ T cells (F). (G, J) Curve graphs show TCR CDR3 aa length distribution of TRA (upper right) and TRB (bottom right) and both(left) in CD4+ T cells (G) and CD8+ T cells (J) across samples pre and post chemotherapy. (TRA: α chains, TRB: β chains, aa: amino acid). (H, K) Violin plots show CDR3 aa length distribution in CD4+ T cells (H) and CD8+ T cells (K). (I, L) Dynamics of dominant CDR3 sequences of TCRs in CD4+ T cells (I) and CD8+ T cells (L). (M) Bar graphs show the fraction of V and J genes in α chains and β chains among T cells. Genes with significant changes are labeled red. (*indicates a FDR < 0.01, ** indicates a FDR < 0.001, *** indicates a FDR < 0.0001, **** indicates a FDR < 0.00001). The results above are generated by comparison between samples (blood_before, blood_after). [file Image_7.tif]

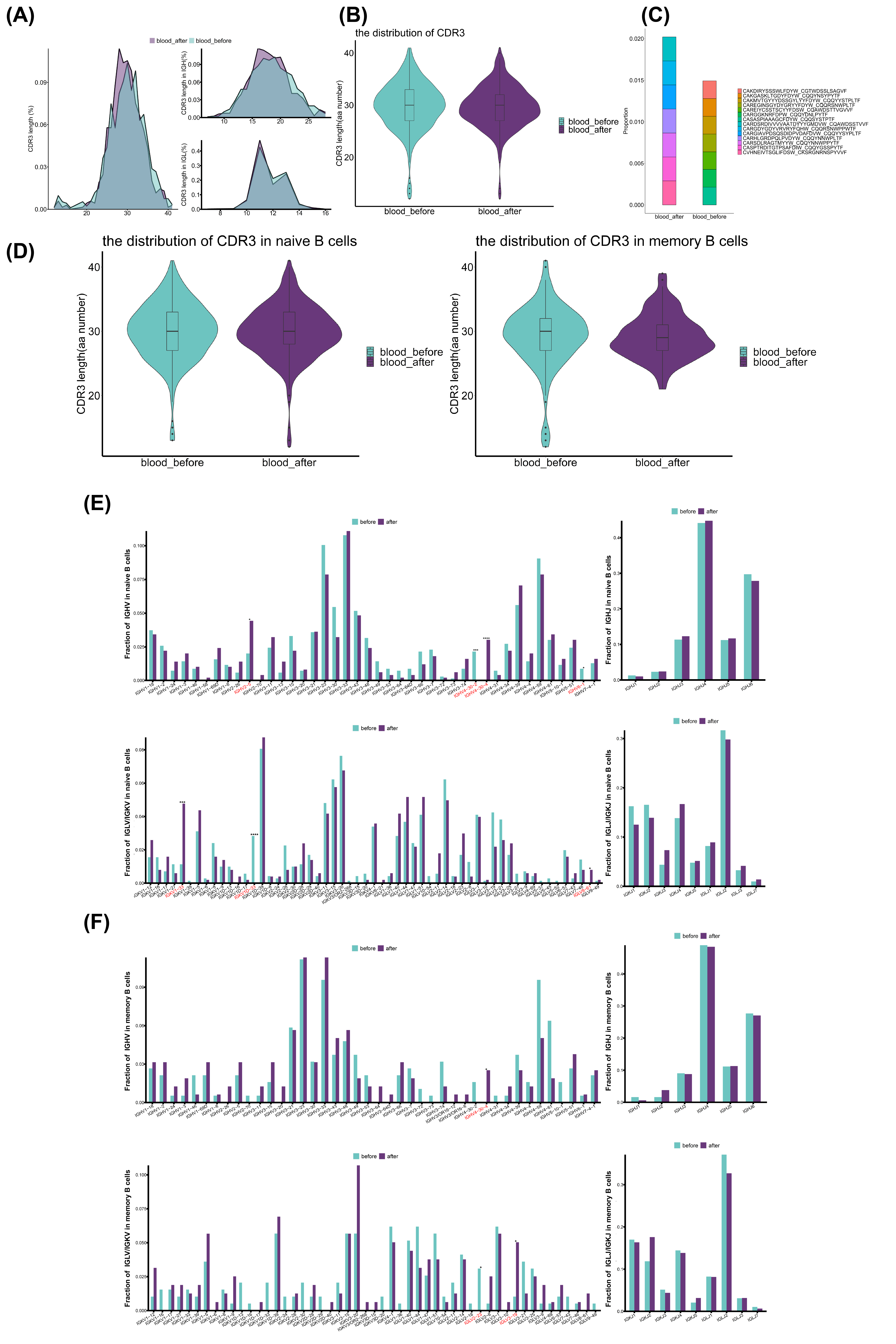

Supplement: Supplementary Figure S8 — Comparative analysis of BCRs in naïve and memory B cells across samples pre and post chemotherapy. (A) Curve graphs show CDR3 aa length distribution of IGL (upper right), IGH (bottom right) and both (left) of all BCRs across samples pre and post chemotherapy. (B) Violin plots show the CDR3 aa length distribution of IGH chains plus IGL chain. (C) Dynamics of dominant CDR3 sequences of BCRs across samples pre and post chemotherapy, colored by types of dominant sequences. (D) Violin plots show distributions of CDR3 length of naïve B cells (left) and memory B cells(right) across samples pre and post chemotherapy. (E and F) Bar graphs show the fraction of immunoglobulin IGHV (upper left), IGHJ (upper right), IGLV/IGKV (bottom left), and IGLJ/IGKJ (bottom right) genes in naïve B cells (E) and memory B cells (F). Genes with significant changes are labeled red. (*indicates a FDR < 0.01, ** indicates a FDR < 0.001, *** indicates a FDR < 0.0001, **** indicates a FDR < 0.00001). The results above are generated by comparison between samples (blood_before, blood_after). [file Image_8.tif]
